# Early oxidative stress and DNA damage in Aβ-burdened hippocampal neurons in an Alzheimer’s-like transgenic rat model

**DOI:** 10.1038/s42003-024-06552-4

**Published:** 2024-07-14

**Authors:** Morgan K. Foret, Chiara Orciani, Lindsay A. Welikovitch, Chunwei Huang, A. Claudio Cuello, Sonia Do Carmo

**Affiliations:** 1 Department of Pharmacology and Therapeutics, McGill University, Montreal, QC Canada; 2 Department of Neurology and Neurosurgery, McGill University, Montreal, QC Canada; 3 Department of Anatomy and Cell Biology, McGill University, Montreal, QC Canada; 4 Department of Pharmacology, Oxford University, Oxford, UK

**Keywords:** Alzheimer's disease, Dementia

## Abstract

Oxidative stress is a key contributor to AD pathology. However, the earliest role of pre-plaque neuronal oxidative stress, remains elusive. Using laser microdissected hippocampal neurons extracted from McGill-R-Thy1-APP transgenic rats we found that intraneuronal amyloid beta (iAβ)-burdened neurons had increased expression of genes related to oxidative stress and DNA damage responses including *Ercc2, Fancc, Sod2, Gsr*, and *Idh1*. DNA damage was further evidenced by increased neuronal levels of XPD (*Ercc2*) and γH2AX foci, indicative of DNA double stranded breaks (DSBs), and by increased expression of *Ercc6*, *Rad51*, and *Fen1*, and decreased *Sirt6* in hippocampal homogenates. We also found increased expression of synaptic plasticity genes (*Grin2b* (NR2B), *CamkIIα, Bdnf, c-fos*, and *Homer1A)* and increased protein levels of TOP2β. Our findings indicate that early accumulation of iAβ, prior to Aβ plaques, is accompanied by incipient oxidative stress and DSBs that may arise directly from oxidative stress or from maladaptive synaptic plasticity.

## Abstract (web summary)

Microdissected amyloid-burdened hippocampal neurons of an Alzheimer-like transgenic rat displayed increased expression of oxidative stress-related genes and DNA damage, which occurred prior to amyloid plaque formation.

## Introduction

The amyloid hypothesis has dominated Alzheimer’s disease (AD) research and clinical trials for decades^1^. Amyloid β (Aβ) initially accumulates intraneuronally (iAβ) as monomers then oligomers−which are the most toxic form^2–5^−prior to extracellular amyloid plaque formation. This accumulation of iAβ results in deleterious effects including synaptic abnormalities^6^, long-term potentiation impairment^7–9^ and cognitive decline^8,10–17^.

Additional mechanisms underlying the AD pathogenesis point towards a role for reactive oxygen species (ROS) and oxidative stress. Aβ can induce oxidative stress through multiple potential mechanisms. Intracellular Aβ has been shown to generate ROS by inserting into cellular membranes and initiating lipid peroxidation through its methionine 35 residue^18–21^. Aβ has also been observed to insert into mitochondrial membranes, decreasing the membrane potential and disrupting mitochondrial function^22–26^. Interactions between Aβ and copper can also indirectly lead to hydroxyl radical production^27,28^. Furthermore, Aβ can bind RAGE (receptor for advanced glycation end products) which activates downstream pathways that indirectly lead to oxidative stress^29,30^. Lastly, Aβ-mediated disruption of NMDA receptor function can result in calcium dyshomeostasis which can lead to oxidative stress^31,32^ and hyperexcitability^33,34^. Of note, neuroinflammation has emerged as an early pathological mechanism in AD which is closely tied to oxidative stress^35,36^.

Although ROS have a physiological role in the brain, including the regulation of synaptic plasticity and memory formation, imbalances in ROS production, antioxidant levels or activity, and redox signaling can culminate in cellular damage, cell cycle reentry^37,38^, and disease through modifications to biomolecules including proteins, lipids, and nucleic acids^39,40^. Neurons are especially vulnerable to oxidative stress and accumulation of oxidative damage due to: (1) high brain O_2_ concentration, (2) the large metabolic demand of neurons influencing mitochondrial ROS production^41,42^ (3) the elevated concentration of polyunsaturated fatty acids in neuronal membranes which are vulnerable to lipid peroxidation, (4) the low ratio of antioxidant to pro-oxidant enzymes in the brain^43,44^, (5) high brain iron content^45^ which can contribute to ROS production via the Fenton reaction^44^, and (6) the reliance on error-prone DNA repair pathways such as non-homologous end joining (NHEJ) in place of replication-associated DNA repair^34,41^.

Oxidative damage in the brain increases with aging^46^ and has been implicated in many neurodegenerative diseases including AD^47^. Markers of oxidative damage have been observed in transgenic animal models of AD^48–50^, as well as the brains of individuals with mild cognitive impairment (MCI)^51,52^, Down Syndrome (DS)^53,54^, and AD^55–57^. Using a proteomic approach, our lab previously showed that cellular stress occurs during pre-plaque stages of the AD-like amyloid pathology^58^.

However, antioxidant clinical trials for AD have not succeeded^59,60^. This suggests that during advanced, late stages of AD, the pathology is irreversible and therefore the opportunity to prevent or delay AD is during the earliest preclinical stages when the initial, disease aggravating oxidative stress occurs. Still, the decades preceding extracellular plaque formation and clinical symptoms remain mostly uncharacterized, thus, understanding the role of neuronal oxidative stress at the earliest stages of AD would offer insight into disease progression.

Studying these early pre-plaque stages is best carried out in reliable models of the AD pathology. We have generated the McGill-R-Thy1-APP transgenic (Tg) rat model exhibiting an AD-like amyloid pathology with a prolonged pre-plaque stage, which allows for studying the effects of gradual iAβ accumulation well before extracellular plaque formation^12,14,36^. Rats are also physiologically, genetically and morphologically closer to humans, with six tau isoforms, similar ApoE properties, similar immune system and a wider behavioral display, compared to mice^61^.

In this study, applying the McGill-R-Thy1-APP rat model, we found increased expression of oxidative stress-related genes in iAβ-burdened hippocampal neurons at pre-plaque timepoints including genes related to DNA damage repair and antioxidant response. This coincided with increased expression of the protein XPD (involved in nucleotide excision repair (NER)), increased double stranded DNA breaks (DSBs), and a trend to increase in 4HNE (4-hydroxynonenal) immunoreactivity in hippocampal neurons. In hippocampal homogenates we found altered expression of DNA repair genes and synaptic plasticity genes and elevated 4HNE immunoreactivity. Overall, the results point towards an incipient oxidative stress response in iAβ-burdened neurons prior to plaque deposition.

## Results

### Pre-plaque, iAβ-burdened hippocampal neurons displayed increased transcript levels of oxidative stress response genes

**Fig. 1:**
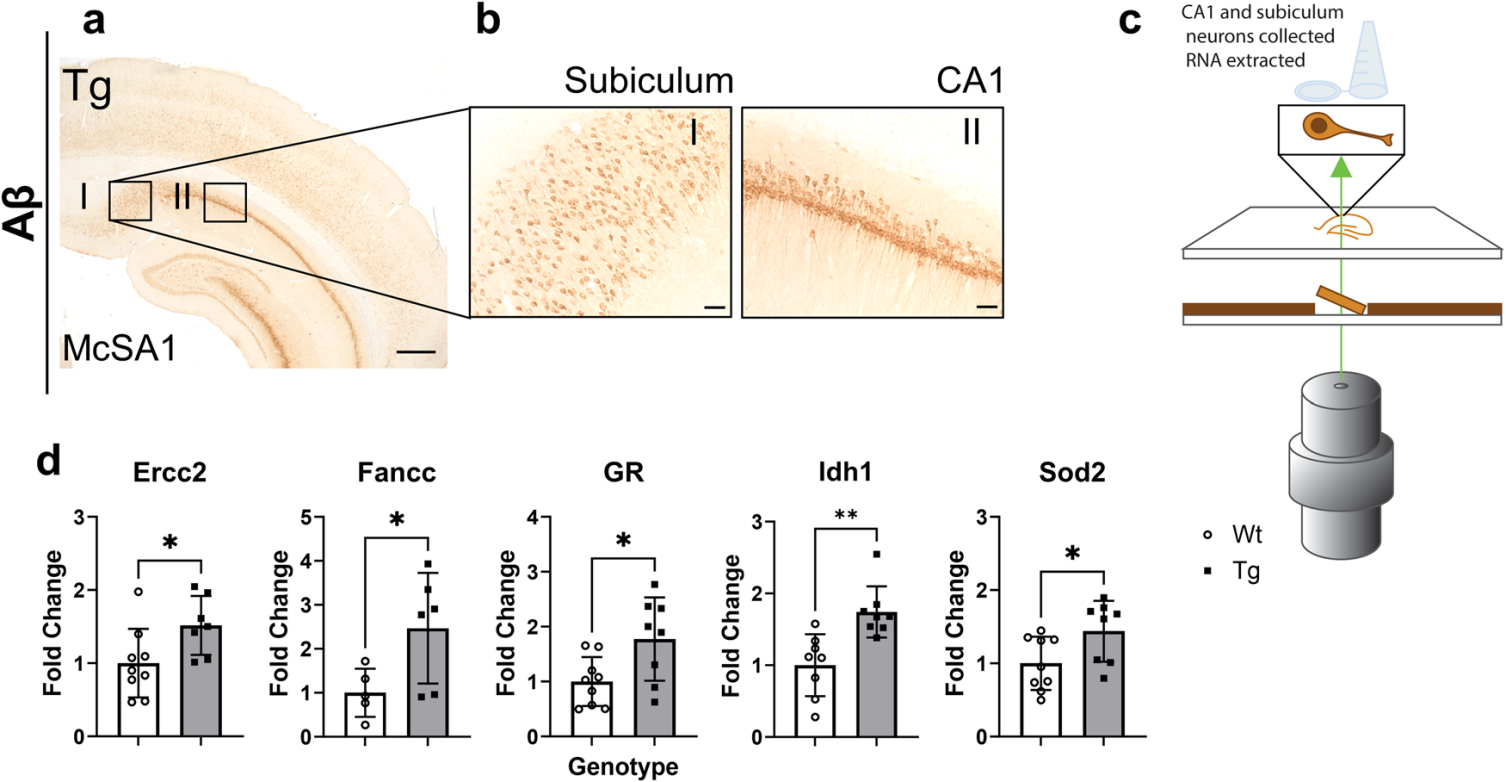
Laser captured iAβ-burdened neurons have increased expression of genes related to oxidative stress and DNA damage response. **a** Representative image of McSA1 (Aβ) immunoreactivity in Tg hippocampus. **b** High magnification McSA1 immunoreactivity in subiculum (I) and CA1 (II) in Tg rats as from A. **c** Schematic depicting laser capture microdissection of CA1 and subiculum neurons from Tg Aβ-burdened neurons and Wt neurons not burdened with Aβ. **d** Differentially expressed genes including *Ercc2*, *Fancc*, *Sod2*, *Gsr (GR)*, and *Idh1* in Aβ-burdened Tg hippocampal neurons as compared to Wt neurons. Fold changes were normalized to Wt expression. Scale bars represent 500 µm in A, 50 µm in (**b**). n = 5–9 (Wt), n = 6–8 (Tg). Error bars indicate SD. two-tailed t-test, *p < 0.05, **p < 0.01.

We investigated the impact of pre-plaque, intraneuronal Aβ (iAβ) accumulation on oxidative stress-related gene expression in hippocampal neurons using laser capture microdissection (LCM) and qRT-PCR (Fig. 1a–c). Pyramidal neurons from CA1 and subiculum were isolated from wild-type (Wt) and Tg McGill-R-Thy1-APP rats, at 5 months of age, a pre-plaque time point where Aβ accumulates within neurons^12^. Intraneuronal Aβ load was confirmed with IHC using the antibody McSA1 which specifically recognizes N-terminal amino acids 1-12 of human Aβ without cross-reacting to APP or its cleavage products^14,62^. McSA1 immunoreactivity showed that hippocampal neurons in Tg rats were burdened with intraneuronal Aβ (Fig. 1a, b) while Wt rats had no immunoreactivity^14,62^. All neuronal RNA samples had a RIN value above 7.0 to ensure quality of extracted mRNA (see^36^ for raw data) and we previously showed that Aβ-burdened hippocampal neurons produce various potent immune factors^36^. In the present study, using the RNA from these laser-captured Aβ-burdened neurons we compared the expression of 84 genes related to oxidative stress between Wt and Tg rats. Five genes, namely, *Ercc2*, *Fancc*, *Sod2*, *Gsr (GR)*, and *Idh1* were significantly upregulated in Tg hippocampal neurons as compared to Wt neurons (Fig. 1d), with four genes (*Ift172*, *Sqstm1*, *Gclm* and *Ercc6*) showing trends to increase in Tg neurons (Fig. S1). In addition to a response to oxidative stress (GO:0006979), gene ontology (GO) and pathway enrichment analyses indicated an ongoing DNA damage response (GO:0006974) in Aβ-burdened neurons (*Fancc, Sod2, Ercc2, Ercc6*).

### DNA repair protein, XPD (*Ercc2*) was increased in transgenic subiculum neurons while Fancc levels remained unchanged

**Fig. 2:**
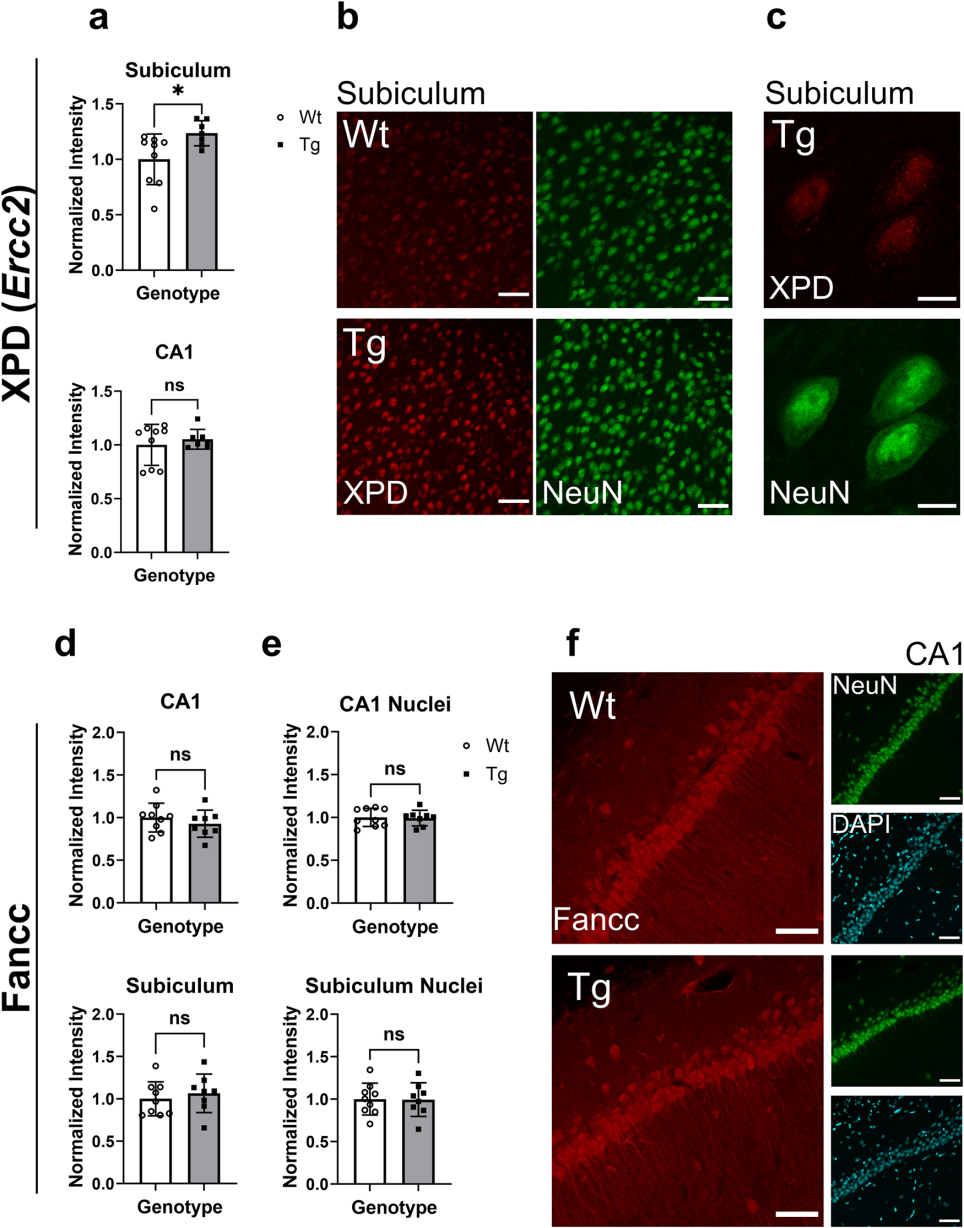
Protein levels of XPD (gene product of *Ercc2*) and Fancc in hippocampal neurons. **a** Quantification of XPD immunoreactivity in subiculum and CA1 neurons of Wt and Tg rats normalized to Wt fluorescence intensity. **b** Representative images of XPD immunoreactivity (red) in Wt and Tg subiculum neurons with NeuN in green. **c** Higher magnification images of XPD immunoreactivity (red) in subiculum neurons with NeuN in green. **d** Quantification of Fancc immunoreactivity in CA1 and subiculum neurons of Wt and Tg rats normalized to Wt fluorescence intensity. **e** Quantification of Fancc immunoreactivity in the nuclei of CA1 and subiculum neurons normalized to Wt fluorescence intensity. **f** Representative images of Fancc immunoreactivity (red) in Wt and Tg CA1 neurons with NeuN in green, and DAPI in cyan. n = 9 (Wt), n = 7–8 (Tg). Error bars represent SD. Scale bars represent 50 µm in B and 10 µm in C. ns = non-significant, two-tailed t-test, *p < 0.05.

To determine whether the changes observed in oxidative stress-related transcripts (Fig. 1d) corresponded to changes at the protein level in these neurons, we performed immunofluorescence labeling beginning with XPD (*Ercc2*) and Fancc which are both involved in DNA damage repair. Consistent with our results at the transcript level, we found elevated levels of XPD in Tg subiculum neurons, an initial brain region affected by Aβ pathology, while levels in CA1 neurons remained unchanged from the Wt (Fig. 2). Regarding Fancc, while RNA levels were upregulated in Tg hippocampal neurons (Fig. 1d), at the protein level, no significant differences were observed. Since Fancc localizes in the cytoplasm and the nucleus, we compared both total and nuclear Fancc levels separately between Wt and Tg hippocampal neurons but did not observe statistical differences (Fig. 2d–f).

### Hippocampal expression of other DNA damage repair genes showed alterations in *Fen1, Ercc6, Sirt6 and Rad51*

In light of our findings in hippocampal neurons, where three of the five upregulated genes (*Ercc2, Fancc* and *Sod2*) were implicated in DNA damage response (GO:0006974) (*Ercc2* in nucleotide excision repair, *Fancc* in interstrand crosslink repair among other repair processes and *Sod2* as a repressor of nuclear genome instability)^63,64^, we investigated the status of other DNA damage repair genes in the Tg hippocampus as compared to Wt. Towards this objective, qRT-PCR of cDNA isolated from hippocampal homogenates was performed.

We found a change in expression of genes that play roles in DNA repair pathways including NER, base excision repair (BER), non-homologous end joining (NHEJ) and homologous recombination (HR). In Tg hippocampal homogenates, there was an increase in expression of the genes *Ercc6* (excision repair cross-complementing 6) involved in BER and NER, *Fen1* (flap endonuclease 1), involved in BER, and NHEJ and *Rad51* (Rad51 recombinase) involved in HR. Conversely, in Tg hippocampal homogenates there was a decrease in *Sirt6* (sirtuin 6), involved in BER, HR, and NHEJ as compared to Wt. Additionally, there was a trend to increase in the genes *Xrcc6* (x-ray repair cross complementing 6, gene product Ku70 involved in NHEJ) and *Ercc3* (involved in NER) (Table 1).

**Table 1:** Quantitative RT-PCR of hippocampal homogenates

Gene	DNA Repair Pathway	Fold-Change Tg	P value
*Ape1*	BER	1.14	0.3854
*Brca1*	HR	1.05	0.8202
*Cdk5*	BER/Others	1.12	0.4985
*Ercc3*	NER	1.21	0.0687***
***Ercc6***	**BER, NER**	**1.42**	0.0450*
***Fen1***	**BER, NHEJ**	**1.31**	0.0136*
*Ogg1*	BER	1.15	0.4558
*Parp1*	Multiple	1.00	0.9973
*Pcna*	Multiple	0.76	0.1994
*Pnkp*	Multiple	1.02	0.5603
*Polβ*	BER	1.10	0.3517
*Prkdc*	NHEJ	1.12	0.4275
*Sirt1*	Multiple	1.24	0.1751
*Sirt3*	mtDNA repair	1.13	0.4735
***Sirt 6***	**BER, HR and NHEJ**	**0.62**	0.0412*
***Rad51***	**HR**	**1.94**	0.0089**
*Rpa*	Multiple	1.27	0.1060
*Tdp1*	Multiple,	1.18	0.2915
*Xrcc1*	BER, NHEJ	1.08	0.6966
*Xrcc4*	NHEJ	0.96	0.5540
*Xrcc5/KU80*	NHEJ	1.05	0.7876
*Xrcc6/KU70*	NHEJ	1.17	0.0634***

*p < 0.05, **p < 0.01, ***trend, text in bold indicates statistically significant changes. n = 12 (Wt), n = 15 (Tg)

### Glutathione reductase protein levels trended to decrease in CA1 neurons of Tg rats while Sod2 and Idh1 levels remained unchanged

**Fig. 3:**
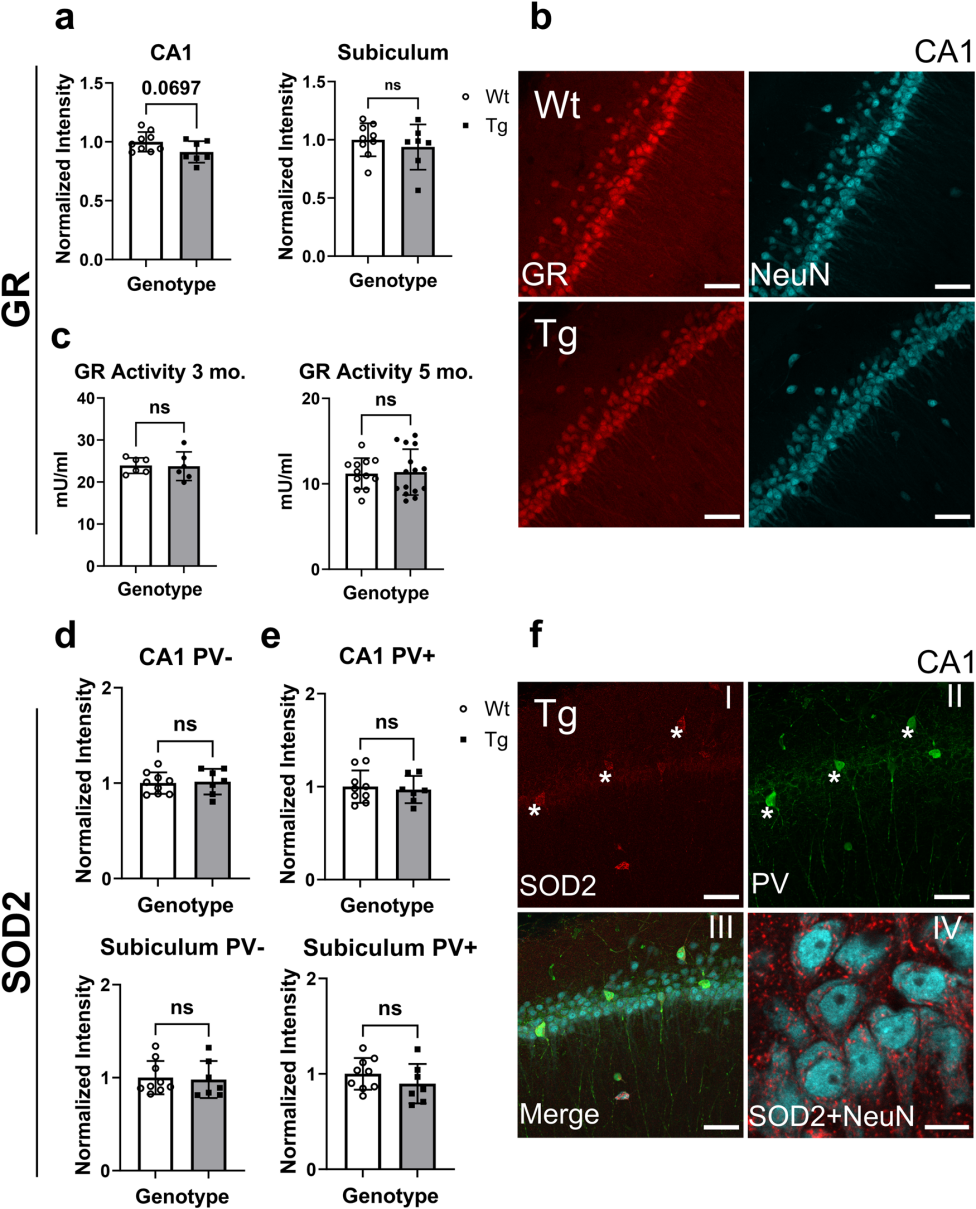
Protein levels of GR and SOD2 in hippocampal neurons. **a** Quantification of GR immunoreactivity in CA1 and subiculum neurons of Wt and Tg rats normalized to Wt fluorescence intensity. n = 9 (Wt), n = 7 (Tg). **b** Representative images of GR immunoreactivity (red) in Wt and Tg CA1 neurons with NeuN in cyan. **c** Enzyme activity of GR in 3-month (n = 6 (Wt), n = 6 (Tg)) and 5-month-old (n = 12 (Wt), n = 15 (Tg)) Wt and Tg cortical homogenates. **d** Quantification of SOD2 immunoreactivity in CA1 and subiculum neurons of Wt and Tg rats that had no parvalbumin (PV-) immunoreactivity. Values were normalized to Wt fluorescence intensity. **e** Quantification of SOD2 immunoreactivity in CA1 and subiculum of Wt and Tg PV+ neurons normalized to Wt fluorescence intensity. n = 9 (Wt), n = 7 (Tg). **f** Representative CA1 images of a Tg rat showing SOD2 immunoreactivity in red (I), PV+ neurons in green indicated by asterisks (*) (II), merged with NeuN in cyan (III). A higher magnification image of SOD2 (red) and NeuN (cyan) in CA1 (IV). Error bars represent SD. Scale bars represents 50 µm in B, F (I-III) and 10 µm in F (IV). ns = non-significant, two-tailed t-tests.

Immunoreactivity for other proteins whose genes were upregulated in hippocampal neurons from Tg rats namely GR, SOD2 and Idh1 (Fig. 1d), was assessed and we found that these proteins were not upregulated as their respective transcripts were. GR was unchanged in the subiculum while there was a trend to decrease in Tg CA1 neurons (Fig. 3a, b), contrary to the increase in transcript levels (Fig. 1d). The activity levels of GR in cortical homogenates were unchanged in 3-month and 5-month-old Wt and Tg rats (Fig. 3c). While for SOD2 mRNA levels were upregulated in Tg hippocampal neurons (Fig. 1d), we did not observe differences at the protein level as assessed by immunofluorescence (Fig. 3d–f). On the other hand, parvalbumin positive (PV + ) neurons displayed higher SOD2 immunoreactivity compared to parvalbumin negative (PV-) neurons in both the CA1 and the subiculum therefore, PV- and PV+ neurons were analyzed separately. However, no significant differences in SOD2 immunoreactivity between Wt and Tg neurons were observed.

Interestingly, hippocampal Idh1 immunoreactivity was more prominently expressed at the protein level in astrocytes rather than neurons. When quantified, immunoreactivity of Idh1 in astrocytes did not differ between Wt and Tg. Furthermore, no differences between Wt and Tg GFAP immunoreactivity were observed (Fig. S2 panel E for analysis details) confirming that increased *Idh1* gene expression in captured neuronal material from Tg hippocampi was not due to increased gliosis as previously demonstrated by Welikovitch *et al*. (2020) utilizing the same laser captured neuronal material, with minimal astrocytic content^36^.

### At pre-plaque stages, transgenic hippocampal neurons showed evidence of incipient oxidative damage

**Fig. 4:**
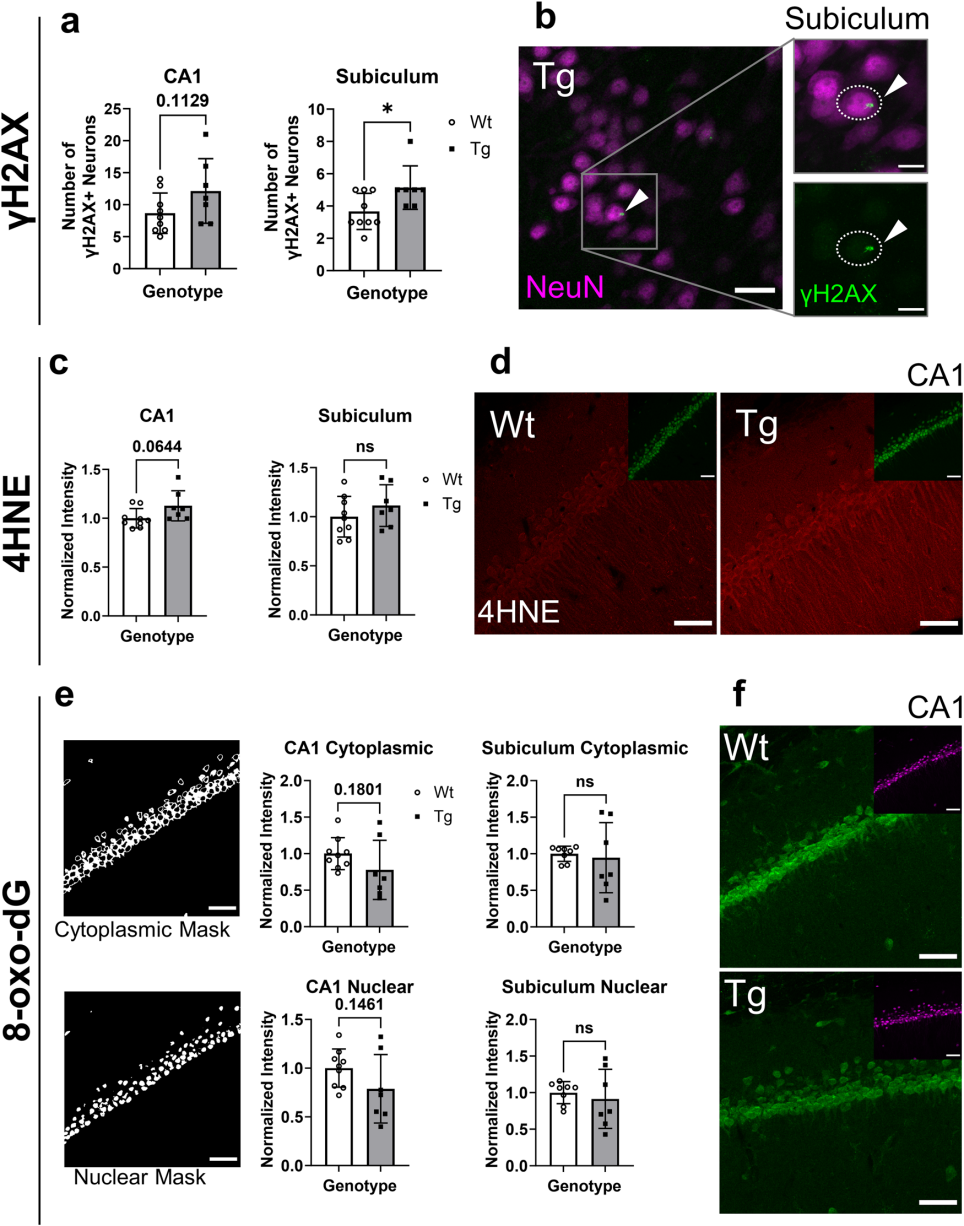
Damage in iAβ-burdened neurons. **a** Quantification of neurons with γH2AX-positive foci in CA1 and subiculum. **b** Representative image of γH2AX-positive foci in green (arrowhead) in the Tg subiculum (NeuN in magenta). **c** Quantification of 4HNE immunoreactivity in CA1 and subiculum of Wt and Tg neurons normalized to Wt fluorescence intensity. **d** Representative images of 4HNE immunoreactivity (red) in Wt and Tg CA1 neurons with an inset showing NeuN in green. **e** Quantification of 8-oxo-dG immunoreactivity in CA1 and subiculum of Wt and Tg neurons normalized to Wt fluorescence intensity. The left-most panels show the cytoplasmic or nuclear masks used to distinguish cytoplasmic versus nuclear immunoreactivity for quantification. **f** Representative image of 8-oxo-dG immunoreactivity in Wt and Tg CA1 neurons (green) with inset showing NeuN (magenta). n = 9 (Wt), n = 7 (Tg). Error bars represent SD. Scale bars represent 50 µm. ns = non-significant, two-tailed t-tests, *p < 0.05.

Given the increased expression of oxidative stress response genes, we examined the extent of downstream oxidative damage in CA1 and subiculum neurons. For this, γH2AX, 4HNE, and 8-oxo-dG immunoreactivity were quantified. Subiculum neurons burdened with iAβ had significantly higher numbers of γH2AX positive neurons (Fig. 4a), while CA1 neurons showed a trend to increased numbers. γH2AX foci indicate the presence of DSBs which can be caused by oxidative DNA damage^65^.

In addition, in hippocampal homogenates, Western blotting revealed a significant increase of 4HNE adduction with protein targets—which reflects levels of lipid peroxidation, a downstream form of oxidative damage (Fig. S3). However, quantification of neuronal 4HNE immunoreactivity by immunofluorescence only showed a trend to increase in CA1 Tg neurons (Fig. 4c, d) suggesting that the neuronal body may not be the only cell component affected by oxidative damage.

Next, oxidized DNA was assessed by probing for 8-oxo-dG. Notably, the antibody used can recognize both 8-oxo-dG and 8-oxo-G, thus, pre-treatment with either DNase or RNase helps to elucidate RNA- or DNA- specific oxidation respectively. As such, pre-treatment of sections with RNase resulted in a decreased immunoreactivity (Fig. S4) and recognition of DNA (nuclear and mitochondrial) rather than RNA oxidation. Nuclear, and cytoplasmic immunoreactivity of 8-oxo-dG in Tg and Wt hippocampal neurons was quantified using immunofluorescence (Fig. 4e, f), and no differences between Tg and Wt neurons were found. However, interestingly, there was a trend to decrease in 8-oxo-dG immunoreactivity in CA1 neurons of Tg animals (Fig. 4e).

The general redox status of 5-month cortical tissue was assessed using an assay employing the fluorescent probe DCF (dichlorodihydrofluorescin) (Fig. S5) and no changes between Wt and Tg cortical homogenates were detected. Of note, interpreting results from this particular probe must be exercised with caution, since it can react with various free radicals in the cell, and it is dependent on peroxidase activity and the availability of free iron among other factors^66^.

### Incipient oxidative stress is associated with increased hippocampal protein levels of TOP2β and expression of early response genes (ERGs)

**Fig. 5:**
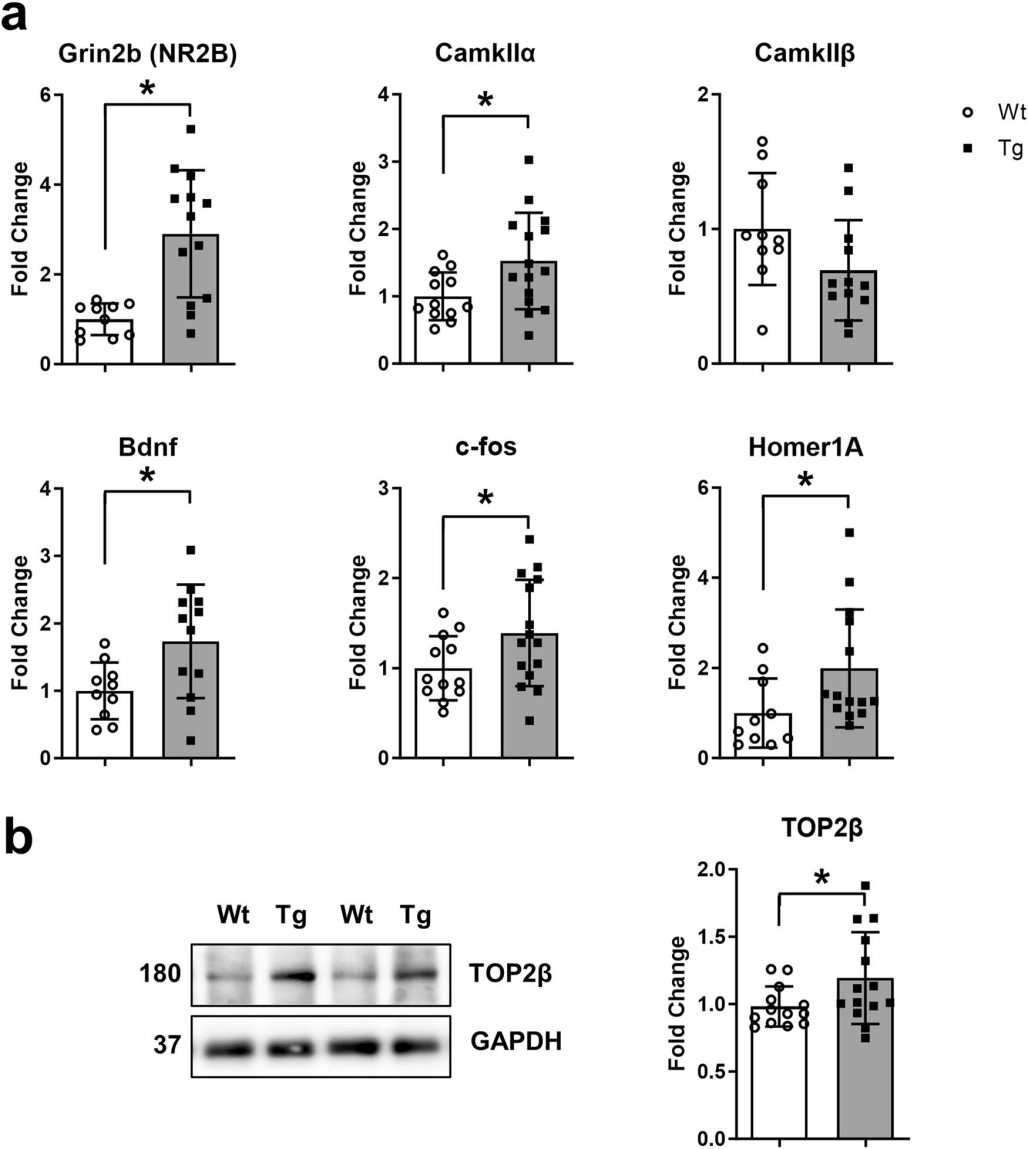
Increased transcript levels of ERGs and TOP2β protein levels in hippocampal homogenates. **a** Differentially expressed synaptic plasticity genes including *Grin2b*, *CamkIIα, BdnfIV, c-fos* and *Homer1A* in Tg hippocampal homogenates as compared to Wt. **b** Representative images (left) and quantification (right) of TOP2β immunoreactivity normalized to GAPDH levels in Wt as compared to Tg hippocampal homogenates as determined by Western blotting. Fold changes were normalized to Wt expression. n = 10–13 (Wt), n = 12–15 (Tg). Error bars indicate SD. Two-tailed t-test, *p < 0.05.

While excessive ROS is associated with decreased performance in cognitive function, physiological concentrations of ROS are necessary to regulate activity-dependent neuronal plasticity^67^. Evidence suggests that neuronal activity triggers the formation of DNA DSBs to initiate rapid transcription of ERGs, (including *Bdnf*, *c-fos*, and *Homer1A*), which are implicated in synaptic plasticity, an action likely mediated by DNA Topoisomerase IIβ (TOP2β)^68^. In Tg hippocampus, we found increased transcript levels of ERGs and increased protein levels of TOP2β compared to Wt (Fig. 5a, b, Fig. S6).

Neuroplasticity is accompanied by activation of Ca^2+^/calmodulin-dependent protein kinases (CaMKIs) (reviewed in Minichiello, 2009)^69^ and activation of NMDA receptors (reviewed in Yamada et al. 2002)^70^. Therefore, we further examined transcript levels of *Grin2b* (encodes N-methyl-D-aspartate receptor (NMDAR) 2B), and the downstream interactor *CamkIIα* which were significantly upregulated in Tg hippocampal homogenates as compared to Wt (Fig. 5a).

## Discussion

In the present study, we demonstrate that iAβ accumulation in 5-month-old McGill-APP transgenic rats induced incipient oxidative stress, DNA damage, and maladaptive expression of synaptic plasticity genes in hippocampal neurons, months before initial Aβ plaque deposition and independent of cell death^71^. Past publications from our laboratory have established that McGill-APP rats display significant deficits in several behavior tasks and in electrophysiology parameters at this age^9,12,14,16,17^.

We have also previously shown by applying proteomic approaches that McGill-APP rats display changes in the expression of proteins related to oxidative stress at pre-plaque stages of the pathology^58^. Importantly, using the same preparation of LCM isolated neurons used in the present study, our laboratory discovered that cytokines and chemokines are produced by these iAβ-burdened neurons before the glial inflammatory response, suggesting that the accumulation of iAβ in neuronal cell bodies also initiates an inflammatory process coinciding with the early oxidative stress described here^35,36,72,73^.

This incipient oxidative stress process was evidenced by increased neuronal transcript levels of oxidative stress response genes (*Sod2*, *Idh1*, *Gsr (GR)*, with trends to increase in genes *Ift172*, *Sqstm1*, *Gclm*) and by increased levels of 4HNE (trend towards an increased 4HNE-IR in Aβ-burdened CA1 neurons and increased levels of 4HNE in hippocampal homogenates). This oxidative stress response coincided with a DNA damage response as evidenced by (1) changed transcript levels of genes involved in DNA repair (increased neuronal expression of *Ercc2, Fancc, Sod2* and increased hippocampal expression of *Ercc6, Fen1, Rad51*, a trend to increase in *Xrcc6* and *Ercc3* and a decrease in *Sirt6*), (2) increased neuronal protein levels of XPD (*Ercc2*), which is involved in nucleotide excision repair (NER), and (3) an increased number of γH2AX nuclear foci in neurons, indicative of DSBs. These processes were accompanied by increased hippocampal transcript levels of synaptic plasticity genes (*Grin2b* (NR2B), *CamkIIα, Bdnf, c-fos*, and *Homer1A*) as well as increased protein levels of TOP2β.

Despite the increased expression of oxidative stress response genes in hippocampal neurons, the methodology applied did not always reveal significant changes in their protein levels. One such example was *Sod2* which showed no changes at the protein level (Fig. 3d–f) despite increased transcripts in Tg neurons (Fig. 1d). However, since SOD2 is a mitochondrial antioxidant enzyme, our analysis at the protein level by IF was limited in that we could not quantify CA1 neuron-specific synaptic levels of SOD2. Indeed, it could be possible that at the level of the synapse, there is SOD2 deficiency in the Tg hippocampus. Other publications have demonstrated that synaptic mitochondrial deficits precede non-synaptic mitochondrial deficits in Tg AD models including heterozygous McGill-R-Thy1-APP rats^24,74^. Interestingly parvalbumin positive (PV + ) neurons expressed significantly higher levels of SOD2, likely due to the increased need for protection against oxidative stress related to increased activity (Fig. 3f qualitative data shown, Fig. S2c, d for method of PV+ and PV- SOD2 quantification by IF)^75^.

Similarly, the increased *Gsr* (*GR*) gene expression in Tg hippocampal neurons only translated into a trend to heightened GR protein levels in CA1 (but not in subiculum). It is noteworthy that despite unchanged levels of antioxidant proteins, the antioxidant capacity of these proteins might be compromised by oxidative modifications^76^. This would be supported by the trend to increase in 4HNE levels in CA1. 4HNE can bind to both proteins and DNA, potentially inactivating certain antioxidant enzymes and further aggravating oxidative stress. However, GR activity levels were found unchanged in cortical homogenates from McGill-APP rats. The unchanged GR activity levels aligns with results from another study that utilized a Tg mouse model of AD^50^. However, the limitation here is that we did not assess hippocampal, nor neuron-specific GR activity which may be of relevance. One study assessing non-cognitively impaired (NCI), MCI and AD brain tissue including synaptosomal and mitochondrial fractions, showed that GR activity was only decreased in MCI and AD synaptosomal fractions^77^, i.e., at later stages.

Glutathione reductase aids in replenishing glutathione (GSH) levels in the cell by catalyzing the conversion of oxidized glutathione (GSSG) back to reduced glutathione (GSH). GSH is a non-protein antioxidant with brain concentrations of 1–3 µM which protects the cell by reacting with free radicals, but also by aiding glutathione peroxidases in breaking down hydrogen peroxide (H_2_O_2_)^78^. Thus, the ratio of reduced to oxidized glutathione (GSH:GSSG) is indicative of the cellular redox state which is altered in aging^79^, in Tg rodent models of AD^50,58^ and peripherally in MCI and AD^80^. Another important function of GSH is to detoxify reactive electrophiles such as 4HNE^81^. In this case, GSH is enzymatically conjugated to 4HNE by glutathione-S-transferases, of which there are many isoforms. However, the increased levels of 4HNE in hippocampal homogenates (which aligns with increases we previously reported in cortical homogenates^82^) but only trend to increase in CA1 4HNE-IR suggests that the major source of 4HNE may not be the neuronal cell body. In line with this, Tg mAPP preparations of synaptic mitochondria showed significantly higher 4HNE levels compared to nonsynaptic Tg mitochondria preparations. This observation occurred in animals as young as 4 months of age, prior to extensive extracellular Aβ accumulation and when Aβ could be detected in synaptic but not in nonsynaptic mitochondria^24^. Another possibility would be that the major source of 4HNE is non-neuronal, however 4HNE immunoreactivity appears to be primarily neuronal.

A key marker of oxidative stress is DNA damage. Oxidative damage to DNA accumulates with aging^83^ and can result in DSBs when there are multiple lesions in proximity to one another (within 20 bp), also known as oxidatively induced clustered DNA lesions^84,85^. Additionally, in association with or independently of oxidative stress, non-dividing cells such as neurons can accumulate DSBs from transcription^68^ but also through abnormal activity and cell cycle reentry^37,38,68,86,87^. When DSBs occur, the histone variant H2AX is rapidly phosphorylated at serine 139 to form γH2AX^88^, which then accumulates at DSBs as foci to help recruit repair proteins^89–91^. These γH2AX positive foci are one of the earliest markers of DSBs and can be visualized using immunofluorescence^34,92^. By immunofluorescence, we found a significantly increased number of neurons with γH2AX positive foci in the Tg subiculum, while Tg CA1 neurons showed a trend to increase compared to Wt neurons (Fig. 4a), indicating that in our model, DSBs are an early consequence of iAβ accumulation, occurring well before Aβ plaque deposition and neuronal loss^71^.

Our findings align with another study reporting an increased number of neurons with γH2AX positive foci in the entorhinal cortex and dentate gyrus of Tg hAPP-J20 mice as early as at 1.5 to 2.2 months of age, prior to plaque deposition and cognitive impairment^86^. Increased numbers of both neurons and astrocytes with γH2AX positive foci were also reported in the hippocampus and frontal cortex of MCI and AD brains^34^. However, a measure of caution is warranted when interpreting the findings of earlier studies of γH2AX in AD brains and AD models since γH2AX foci are indicative of DSBs, while pan-nuclear immunoreactivity is indicative of neuronal activity, as highlighted by Shanbhag et al. (2019)^34^.

The accumulation of DSBs in iAβ-burdened neurons appeared to occur independently of oxidative lesions to DNA given that 8-oxo-dG levels remained unchanged between Wt and Tg hippocampal neurons, while a trend to decrease in CA1 Tg neurons was observed (Fig. 4e, f). This contrasts with the increased levels of oxidatively modified neuronal DNA previously reported in post-mortem material from individuals with DS^93^, pre-clinical AD (PCAD)^94,95^, MCI^96^, and AD^97–102^. However, it remains unknown whether this oxidative damage to nucleic acids plays an early role in the AD pathology.

In response to DNA damage, complex signaling pathways are activated to elicit DNA repair. Alterations in expression and activity of DNA repair genes and proteins have been observed at late stages in the AD pathology in the brains of individuals with MCI, and AD^65,103–105^, (reviewed in Bucholtz and Demuth (2013))^106^, and in transgenic rodent models of AD^65,107,108^. In the present study, during pre-plaque stages, iAβ-burdened hippocampal neurons revealed increased expression of *Ercc2* and *Fancc* which are both implicated in DNA repair processes, namely nucleotide excision repair (NER) and interstrand crosslink repair, respectively^109^. However, *Fancc* may also play a role in other repair pathways^110^, response to oxidative DNA damage^111^, and the redox state of the cell^112^. Increased expression of *Fancc* has been reported in astrocyte- and oligodendrocyte-enriched fractions of AD brains at the single-nuclei level^113^. Although *Fancc* may be primarily expressed by glia at later AD stages, our IHC experiments indicate that *Fancc* expression is prominent in neurons at these earlier pre-plaque stages. This finding is in line with our previous report, of a pro-inflammatory process starting in Aβ-burdened neurons before becoming almost exclusively glial at later stages^36^.

Additionally, XPD (Xeroderma pigmentosum complementation group D protein), the gene product of *Ercc2* was elevated in subiculum neurons (Fig. 2). XPD is an ATP-dependent 5’-3’ helicase, that plays a role in RNA polymerase II initiated transcription and in NER^114^. NER acts on a variety of bulky DNA lesions including those resulting from oxidative damage^115^. Notably, previous studies have shown that *Ercc2* gene expression was increased in the brains of individuals with Down Syndrome (DS)^116^, while XPD protein expression was increased in the brains of individuals with DS and AD^117^. Importantly, individuals with DS develop AD due to triplication of chromosome 21 which contains the *APP* gene, causing excessive Aβ production. As a result, individuals with DS exhibit progressive brain Aβ accumulation from as early as birth^118–121^.

The investigation of additional genes in hippocampal homogenates further highlighted impairments in multiple DNA repair pathways (Table 1) including BER, NER and, most importantly, NHEJ and HR, which can repair DSBs^122^. As such, we found an increase in *Ercc6*, which encodes the protein CSB. Like XPD, CSB plays a role in NER but also contributes to base excision repair (BER) which is responsible for repairing a wide variety of oxidative DNA damage^123,124^. Alterations in CSB levels have been linked to neurodegeneration but not directly to AD. There was also a significant increase in the expression of *Fen1* (flap endonuclease 1), which plays a role in BER^125^ and non-homologous end joining (NHEJ)^126^ and in *Rad51*, involved in HR^127–129,65^ but their role in AD remains to be determined.

In contrast, *Sirt6*, whose gene product is an NAD^+^-dependent deacetylase and ADP-ribosyltransferase^130^ showed decreased expression in Tg hippocampal homogenates. Sirt6 is one of the earliest factors recruited to DSBs where it promotes the recruitment of other DNA repair proteins including 53BP1 and BRCA1^131,132^. Our findings are in line with other reports showing a decrease in Sirt6 protein expression in transgenic mice (5XFAD)^133^ as well as decreased gene and protein expression in AD patients^133,134^.

Moreover, *Ercc3* and *Xrcc6* trended to increase in expression in Tg hippocampal homogenates. *Ercc3* produces the protein XPB which has ATPase activity and, like XPD (gene product of *Ercc2*), XPB (*Ercc3*) plays a role in NER^135^. Increased gene and protein expression of XPB (*Ercc3*) was found in the brains of individuals with DS^116^ and AD^117^. A recent study in AD postmortem brains also showed that abnormal phosphorylation of KU70 (product of *Xrcc6*) prevents KU70 accumulation at DSB lesions thereby impairing DNA repair^136^. Lastly, although the following genes are implicated in AD at late stages, we did not find differences between Wt and Tg expression of *Ape1*^137,138^, *Cdk5*^137,139–143^, *Parp1*^105^, *Pcna*^144,145^, *Polβ*^146,147^, or *Sirt3*^148–150^,. This is consistent with the activation of DNA repair processes by the earliest iAβ pathology months before the presence of a full-blown AD pathology.

As stated above, in addition to oxidative stress, DSBs in neurons may arise from increased transcription^68^, abnormal activity and cell cycle reentry^37,38,68,86,87^. Accordingly, our studies revealed that the iAβ burden unleashed incipient oxidative stress and increased DNA damage repair mechanisms which are accompanied by an increased expression of genes related to synaptic plasticity. Although seemingly counter intuitive in a context of impaired cognitive performance, this observation is in line with previous observations from our group and others. McGill-R-Thy1-APP transgenic rats display progressive cognitive deficits^12,14–16,151–154^ and impairments in LTP^9,155^ which have been documented as early as at 3 months of age in the absence of amyloid plaques. This early decline in cognitive capabilities is accompanied by a transient increase in transcript levels of genes relevant to synaptic plasticity, learning, and memory processing in the hippocampus, at pre-plaque stages of the Aβ pathology^151^, prior to their decline at later stages of the pre-plaque iAβ pathology^16,151^. Such cognitive decline is not observable in the human species due to cognitive reserve^156^. This observation is also supported by functional MRI (fMRI) studies showing that patients with early-stage mild cognitive impairment (MCI) display an hyperactivation of medial temporal lobe and hippocampal circuits during memory tasks, possibly reflecting inefficient compensatory activity^157–163^ which has also been referred to as excessive and maladaptive synaptic plasticity. This hyperactivation then decreases at later stages of MCI and in AD dementia, to result in hypoactivation of these brain regions. However, such hyperactivation of brain circuits is associated with poorer, not better cognitive performance in individuals with MCI but also in non-cognitively impaired ApoE4 carriers, in young Down syndrome individuals^164^, in aged individuals^165^ and in aged rats^166^.

Furthermore, it has been evidenced that both ROS and Aβ, at physiological levels, can act as second messengers contributing to ‘Hebbian’ synaptic plasticity^67,167–174^. Low, physiological levels of ROS contribute to synaptic plasticity processes in several areas of the nervous system, including the hippocampus, cerebral cortex, spinal cord, hypothalamus, and amygdala. ROS production is also necessary for hippocampal LTP formation^172,175^. Similarly, it has been demonstrated that Aβ plays a role in regulating synaptic function and memory consolidation by regulating the responsiveness of glutamatergic and cholinergic synapses in the hippocampus^176^. We have also shown in vitro that low, physiological levels of iAβ stimulates the activity dependent CRE-directed gene expression through a Rap1/MEK/ERK pathway, a mechanism that should favor synaptic plasticity^10,174,176,177^. In such context, the co-occurrence of iAβ, incipient oxidative stress, increased DNA damage and increased ERGs expression is intuitive. Indeed, DNA DSBs represent an important step in NMDAR signaling pathway activation following neuronal activity stimulation. They dissolve the topological constraints to enhancer-promoter interactions, therefore facilitating the rapid transcription of ERGs, an action likely mediated by TOP2β^68,178^.

TOP2β is a type IIA topoisomerase^179^ which critically regulates the activity-dependent transcription of genes related to autism, cognitive function, neuronal early-response and neuronal survival^68,179–182^. Endogenous expression of TOP2β^68^ is essential for cell survival by promoting DNA repair processes including DNA DSBs^183^ resulting from oxidative insults^183^. The role of TOP2β in the pathogenesis of AD remains to be elucidated^184^. However, decreased levels of TOP2β were reported in primary cerebellar granule neurons incubated with fibrillar Aβ1-42 peptides^185^. As well, downregulation^186^ of TOP2β induced neurodegenerative effects in a cellular model of Parkinson disease.

Several questions arise from this research: (1) How do these processes evolve into the well-documented buildup of oxidative stress and DNA damage markers at late stages of AD? (2) Do these processes play the same role at both ends of the disease spectrum? Notably, the association between oxidative stress and AD is well-established^47,187,188^. Increased oxidative stress in combination with lowered antioxidant defense serves as a promoter of cellular dysfunction and damage in AD and it is well represented in AD animal models. It has also been suggested that oxidative damage is an early event in AD that decreases with disease progression^189–193^. A study demonstrated a significant inverse relationship between the levels of neuronal 8-hydroxyguanosine immunoreactivity and the extent of Aβ burden^189^ that was found in post-mortem brain tissue from clinically and pathologically confirmed cases of AD^189^. The causal relationship between oxidative damage and neurodegeneration in AD has been further supported by redox proteomic analyses revealing oxidative alteration of proteins as early as at the MCI stage in both post-mortem brain tissue and CSF^47,52,57,76,188,191,194–197^. More recently, it has also been shown that infusion of oxidizing agents into the hippocampus of wild type mice was sufficient to trigger Aβ production^192^. Still, information on when oxidative processes are initiated and how they evolve in AD pathogenesis is limited. Examining the initiation and evolution of oxidative stress in AD is further complicated by the fact that detection of oxidative damage is affected by postmortem intervals and the presence of co-morbidities including brain microvascular pathologies. Therefore, while the present study provides valuable insights on the early processes triggered by intracellular Aβ accumulation, further studies are warranted to acquire a more complete understanding of the disease’s pathology and ultimately define potential interventions. These should include: (1) Examination of these processes at different stages of the disease, (2) mechanistic studies to pinpoint signaling pathways involved in the regulation of the oxidative stress and DNA damage response markers and (3) studies addressing whether the deficits reported here can be reverted with antioxidant treatments.

In summary, the present study revealed that early accumulation of iAβ, coinciding with a neuron-derived inflammatory response (as previously shown^36^) also coincides with altered expression of oxidative stress-related genes including key DNA repair and antioxidant genes, where not all gene level alterations were reflected at the protein level. These modest changes are likely in response to reactive oxygen species (ROS) production and an incipient, but not fully developed, redox imbalance. Furthermore, the lack of overt oxidative damage in these iAβ-burdened hippocampal neurons suggests that this pre-plaque timepoint precedes overt oxidative stress as is observed at late, post-plaque stages of AD. This evidence would favor the argument that an oxidative stress mechanism leading to DNA damage is already present at early, pre-plaque, stages. The DNA damage (DSBs) may arise directly from a combination of oxidative stress and excessive or aberrant synaptic plasticity, through the action of TOP2β. Of interest, our lab has previously shown, at the same pre-plaque timepoint, an excessive hypomethylation in hippocampal neurons was linked to iAβ accumulation^198^.

## Materials and methods

### Animals and tissue collection

Animal work was approved by the McGill Animal Care Committee and followed guidelines established by the Canadian Council on Animal Care (CCAC). We have complied with all relevant ethical regulations for animal use. McGill-R-Thy1-APP Tg rats overexpressing the human *APP* cDNA transgene with both the Swedish and Indiana mutations under the murine Thy1.2 promoter, and their wild-type (Wt) littermates were used for this study^12^. Rats (male and female) were housed in humidity-controlled and temperature-controlled rooms with 12 hour light/dark cycles and given *ad libidum* access to food and water. At 5 months of age, at a pre-plaque stage, rats were deeply anesthetized with intraperitoneal injections containing a mix of chloral hydrate and sodium pentobarbital (6.5 mg chloral hydrate and 3 mg sodium pentobarbital per l00 g body weight), then transcardially perfused with ice-cold saline solution (pH 7.4) for two minutes.

Brains were extracted, and one hemisphere was either: (1) flash frozen in isopentane over dry ice and stored at –80 ⁰C for laser capture microdissection (LCM) experiments or (2) dissected and snap frozen (hippocampus, cortex, cerebellum) then stored at –80 ⁰C for biochemistry experiments. The other hemisphere was post-fixed at 4 ⁰C in 4% paraformaldehyde (PFA) (in 0.1 M phosphate buffer, PB) for 24 h, then saturated with 30% sucrose (dissolved in 0.1 M PB) and coronally sectioned at 40 µm using a freezing sledge microtome (Leica, SM 2000R, Germany). Sections were stored in cryoprotectant solution (37.5% v/v ethylene glycol, 37.5% w/w sucrose in phosphate-buffered saline (PBS)) at –20 ⁰C, pH 7.4 until used for IHC experiments.

### Laser Capture Microdissection and RNA Isolation

Brain tissue that was flash frozen in isopentane over dry ice was sectioned at 10 μm using a Leica CM3050S cryostat and thaw-mounted onto 1.0-mm PEN membrane-covered glass slides that were irradiated for 30 minutes with UV light (MembraneSlide 1.0 PEN; Carl Zeiss). Sections were dehydrated at −20 ⁰C for 30 minutes and stored at −80 ⁰C for later use. Mounted sections were immersed in 95% ethanol, rehydrated using decreasing ethanol concentrations, stained with Cresyl violet for 1 minute, and finally dehydrated with increasing ethanol concentrations followed by xylene. Laser capture microdissection (LCM) was performed after Cresyl violet staining where the pyramidal layer of CA1 and subiculum was collected from 40 tissue sections per animal using the PALM MicroBeam (Carl Zeiss). UV laser settings were: 75 cut energy, 70 cut focus, 12 auto-LPC dot-size. Neurons were identified by diffuse Cresyl violet staining and collected in PCR tubes with an opaque adhesive cap, collection tubes were changed every 2 hours (AdhesiveCap 200 Opaque; Carl Zeiss). Microdissected neuronal samples were incubated with RLT lysis buffer (RNeasy Mini kit, Qiagen) for 30 minutes and RNA was extracted using a RNeasy Mini kit (Qiagen) then stored at −80 ⁰C for later use. To confirm the neuronal enrichment of the isolated mRNA, cDNA was used to measure the relative expression of neuron-specific MAP2 (microtubule associated protein 2) and TUBB3 (βIII-tubulin), microglia/macrophage-specific Iba1 (ionized calcium binding adaptor molecule 1) and CD13, astrocyte-specific GFAP (glial fibrillary acidic protein), and oligodendrocyte-specific MBP (myelin basic protein) transcripts, using the ΔΔCT method. The housekeeping genes were as follows: ACTB (β-actin), CYC1 (cytochrome c 1) and RPL13 (60 s ribosomal protein L13) as reported in ref. ^36^.

### RT^2^ rat oxidative stress profiler PCR array

The quality of RNA isolated from microdissected neuronal material was verified using an RNA 6000 Pico Kit and an Agilent 2100 Bioanalyzer (Agilent Technologies, USA), whereby all samples resulted in an RNA Integrity Number (RIN) higher than 7.0^36^. Isolated RNA was converted to cDNA using the RT^2^ PreAMP cDNA Synthesis Kit and amplified using RT^2^ Rat Oxidative Stress PreAMP Pathway Primer Mix (PBR-065Z, Qiagen). Expression of 84 oxidative stress-related genes was assessed by qRT-PCR (50 thermo cycles total) for each animal using the RT^2^ Rat Oxidative Stress Profiler PCR Array (PARN-065ZD, Qiagen), a CFX Connect Real Time cycler (Bio-Rad) and cycle conditions recommended by the manufacturers. Relative expression of each gene was calculated by the ΔΔC_T_ method, standardized with five housekeeping genes and using the recommended control values from the RT^2^ PreAMP cDNA Synthesis Handbook, where C_T_ values above 35 were considered a negative call. As part of the RT^2^ Profiler PCR Array, three internal controls were included: a genomic DNA contamination control, a reverse transcription control, and a positive PCR control. See Table S1 for the housekeeping genes used in the RT^2^ Rat Oxidative Stress PCR Array and Table S2 for expression results of all genes in hippocampal neurons.

### Immunohistochemistry

#### Brightfield Immunohistochemistry

PFA-fixed free-floating, 40 µm coronal brain sections were washed with PBS to remove cryoprotectant. Then, endogenous peroxidase activity was quenched using a solution of 3% H_2_O_2_, and 10% methanol in PBS for 30 minutes. Following washes with PBS and then with PBS containing 0.2% Triton-X-100 (PBS-T), sections were blocked for 1 hour at room temperature (RT) in 10% normal goat serum (NGS) in PBS-T. Sections were incubated with primary antibodies (Table S3) overnight at 4 ⁰C in 10% NGS: anti-Aβ (McSA1, 1:1000, Medimabs, Canada)^62^. Sections were then washed with PBS-T and incubated with rabbit-anti-mouse (produced in-house, Table S4) (1:25) for 1 hour RT. After washing, sections were incubated for 1 hour with mouse anti-horseradish peroxidase (1:30) that was pre-incubated for 30 minutes with horseradish peroxidase (HRP) (5 μg/ml, 1:200) (MAP kit, Medimabs, Canada). Sections were then washed, and the staining was developed using 0.06% of 3,3′-diaminobenzidine (DAB) (Sigma-Aldrich, Germany) and 0.02% H_2_O_2_ to initiate the reaction. Sections were mounted on pre-cleaned Super Frost (Fisher) gelatin-coated slides, air-dried, dehydrated using increasing ethanol concentrations, cleared with xylene and coverslipped with #1.0 coverslips and Entellan (EM Science, USA).

#### Immunofluorescence

PFA-fixed free-floating, 40 µm coronal brain sections were washed using PBS to remove cryoprotectant. For certain primary antibodies (namely: anti-Fancc, anti-γH2AX, anti-Idh1, anti-Sod2, and anti-XPD) sections underwent heat-mediated antigen retrieval and were incubated at 80 ⁰C in 10 mM citrate buffer (pH 6.0) for 30 minutes. After 20 minutes of cooling at RT, sections were washed using PBS and the standard protocol was resumed. Sections were permeabilized using 50% ethanol for 20 minutes, washed with PBS-T, and blocked for 1 hour at RT in 10% NGS. Sections were incubated with primary antibodies (Table S3) overnight at 4 ⁰C in 5% NGS. After primary antibody incubation, sections were washed with PBS-T and incubated with varying combinations of Alexa Fluor 488 (goat-anti-mouse), Alexa Fluor 568 (goat-anti-rabbit), and/or Alexa Fluor 647 (goat-anti-guinea pig) (all at 1:800, Thermo Fisher Scientific) for 2 hours RT (Table S4). Following washes, to reduce autofluorescence, sections were incubated for 5 minutes with 0.3% Sudan black in 70% ethanol. Sections were then washed three times for 5 minutes each in PBS-T, then three times for 5 minutes each in PBS. In some experiments, sections were then incubated with DAPI (0.1 µg/ml) for 5 minutes and washed with PBS. Sections were then mounted on pre-cleaned Super Frost (Fisher) gelatin-coated slides and coverslipped with #1.5 coverslips and Aqua-Poly/Mount (Polysciences). Note that for γH2AX experiments TBS and TBS-T (0.5% triton-X-100) were used instead of PBS. Negative control experiments including application of secondary antibody alone (no primary) and primary alone (no secondary) were performed. Similarly, as part of our control experiments for antibody selection, the primary 4HNE antibody was pre-adsorbed with blocking peptide (Abcam, ab194193) at antigen to antibody ratios of 0:1, 2:1, 5:1, 10:1 overnight at 4 °C.

### Microscopy and image analysis

#### Brightfield Imaging

For brightfield imaging of McSA1 (Aβ) immunolabelling, an Axio Imager M2 microscope with an AxioCam 506 color digital camera, and ZEN Imaging software (ZEN Blue; Zeiss, Germany) were used. Objective lenses with 2.5x and 20x magnification were used to acquire images of CA1 and subiculum, z-stacks were imaged and collapsed into a single plane image for representation of amyloid beta immunoreactivity.

#### Fluorescence imaging

Confocal images were acquired using an LSM800 Confocal Microscope AxioObserver (Zeiss, Germany) and a 20X Plan Apochromat objective lens (NA = 0.80) with ZEN Imaging software (ZEN Black). To allow quantitative comparisons, images were acquired with the same microscope settings, adjusted specifically for each marker assessed. Z-stacks from 2–3 sections per animal were acquired for CA1 (three image regions) and the subiculum (one image region) with intervals of either 1 µm or 2 µm as determined by the marker of interest. Depending on the fluorophores in each experiment, diode lasers of 405, 488, 561, and/or 640 nm were imaged sequentially from longest to shortest wavelength, all with a pinhole size equivalent to 1 airy unit (AU) for each respective wavelength. 16-bit images (312.5 × 312.5 µm) were acquired with a pixel dwell of 0.76 µs and an averaging of four by line (1 pixel = 0.31 µm). Signal was detected using a Gallium arsenide phosphide (GaAsP) PMT with emission wavelengths of 450-495 nm (405 laser), 500-550 nm (488 laser), 575-650 nm (561 laser no 647 fluorophore), 571-620 nm (561 laser with 647 fluorophore), 650-700 nm (640 laser). To quantify γH2AX+ neurons, five images of CA1 and two images of the subiculum per section (two sections per animal) were acquired using a 20X Plan Apochromat objective (NA = 0.80) with 1 pixel = 0.21 µm. Qualitative images at higher magnifications were acquired using a 63X Plan Apochromat (NA = 1.40) oil immersion objective (pixel = 0.05 µm).

#### Image Analysis

Custom, automated ImageJ macros were created for each target investigated (Fig. S2). Briefly, regions of interest (as example CA1 pyramidal neurons) were identified by the NeuN channel since NeuN specifically identifies mature neurons^199^ and a mask/region of interest (ROI) was generated using this channel. We then quantified immunoreactivity of each protein of interest in areas overlapping with NeuN immunoreactivity. This was followed by quantification of signal intensity using a sum of the z-stack in the channel of interest, thus avoiding any bias in mask/ROI generation. We adapted our experiments to include DAPI labeling for nuclei when cellular localization of the protein needed to be considered, as example, to compare nuclear versus cytoplasmic protein levels (Fig. S2). However, for Idh1 quantification in astrocytes (data not presented), the Idh1 immunoreactivity was used to generate a mask for ROI selection since GFAP and S100β did not capture the entire Idh1 immunoreactive areas (Fig. S2).

Background corrections were performed for XPD, Fancc, GR, 8-oxo-dG and Idh1 as follows: (1) two regions of interest (ROIs) were selected from the summed z-stack (50 ×50 pixels in dimension and in areas of tissue coverage), (2) these intensity values were divided by area (accounting for number of z-stacks) and then averaged generating a mean background value. This value was then subtracted from the intensity measurement generated from neuronal areas of interest. SOD2 and 4HNE were not background corrected since areas lacking immunoreactivity were not reliably found (e.g.,: SOD2 localizes to mitochondria which are widespread in the hippocampus, and 4HNE is generated by lipid peroxidation which is associated with lipid membranes that are also widespread in the hippocampus).

### Glutathione reductase assay

Following the Glutathione Reductase (GR) Assay Kit (ab83461, Abcam), twenty micrograms of cortical tissue from Wt and Tg animals was homogenized in 200 μl of assay buffer on ice then sonicated twice in the span of five seconds and centrifuged at 10,000 g for 15 minutes (4 ⁰C). Protein concentration of the supernatant was quantified using a Lowry assay and each sample was aliquoted and diluted to a total of 100 μl at 5 μg/μl. The samples were then pre-treated with 3% H_2_O_2_ for 5 minutes at RT followed by catalase to stop the reaction. Samples were then added in duplicates (60 μg/well determined through pilot experiments) to a 96-well plate along with the appropriate TNB standard and positive control as provided in the kit. Reduced glutathione (GSH) reacts with 5,5’-Dithiobis (2-nitrobenzoic acid) DTNB in the reaction mix to generate TNB which has an absorbance maximum at 405 nm. The reaction mix was then added to all sample wells and the absorbance (OD_450_) was measured every minute for 60 minutes. Glutathione reductase activity was calculated using the linear range of the curve with T_1_ at 1 minute and T_2_ at 15 minutes. First, the baseline absorbance was subtracted (T_0_ which preceded the addition of the reaction mix) and then these corrected absorbance values at T_1_ and T_2_ were used to calculate ΔA_405nm_ = A_2_ – A_1_. This absorbance value (ΔA_405nm_) was applied to the TNB standard curve to obtain ΔB (the change in TNB concentration in nmol). The below equation was then used to calculate the mU/mL of GR activity where V represents the amount of sample added per well:

$${GR}\; {Activity}=\,\frac{\varDelta B}{\left({T}_{1}-{T}_{2}\right)\times 0.9\times V}\times {Sample}\; {dilution}\; {factor}={mU}/{mL}$$

### DCF assay

The DCF ROS/RNS fluorogenic Assays (Abcam, ab238535) was used to determine the levels of ROS and RNS by measuring the fluorescence intensity in cortical extracts from Wt compared to Tg rats. The assay applies a fluorogenic probe, dichlorodihydrofluorescin DiOxyQ (DCFH-DiOxyQ), which is based on similar chemistry to 2’, 7’-dichlorodihydrofluorescein diacetate. 10–20 mg of cortical tissue was homogenized in 20 volumes of PBS by sonication on ice. Insoluble particles were removed by centrifugation at 10,000 g for 5 min. Supernatants were used to perform the assay following the manufacturer’s instructions. The DCFH-DiOxyQ probe was added to the supernatants in the presence of the catalyst for 30 min and then fluorescence intensity was measured at ex/em 480/530 nm using a Synergy 2 (Bio Tek Instruments, USA). Concentration of H_2_O_2_ in the sample was calculated from the H_2_O_2_ standard curve in µM and normalized by the protein content.

### Quantitative PCR of hippocampal homogenates

#### RNA isolation and cDNA synthesis

Fifteen to twenty mg of cortical tissue was cut for RNA extraction using the RNeasy Mini Kit (Qiagen, 74104) following the manufacturer’s instructions. Quality of isolated RNA was confirmed by obtaining RNA Integrity Numbers (RIN) using a RNA 6000 Pico Kit and an Agilent 2100 Bioanalyzer (Agilent Technologies), all samples had RINs higher than 7.0. To synthesize cDNA, 500 ng of RNA was used for reverse transcription using iScript Reverse Transcription Supermix (Bio-Rad, 1708841) according to the manufacturer’s instructions with the thermal cycle as follows: 5 minutes at 25 ⁰C, 20 minutes at 46 ⁰C and 1 minute at 95 ⁰C.

#### Quantitative real-time PCR

Quantitative real-time PCR was performed using a total reaction volume of 10 μl, containing 2 μl of diluted cDNA, SsoAdvanced Universal SYBR Green Supermix (1x) (Bio-Rad), and a final concentration of 0.25 μM or 0.5 μM of forward and reverse primers (designed using Primer-BLAST, Table S5), with a CFX Connect Real-Time Cycler and CFX manager (Bio-Rad). Cycling conditions were as follows: 30 seconds at 95 ⁰C, then 40 cycles of 10 seconds at 95 ⁰C, 30 seconds at 60 ⁰C followed by a melt curve from 65 ⁰C to 95 ⁰C at 0.5 ⁰C intervals. Gene expression fold change was quantified using the 2^(-ΔΔCT)^ method with HPRT and GAPDH as housekeeping (control) genes (see Supplementary Table S5 for primer sequence details).

### Western Blotting of Hippocampal Homogenates

Cortical tissue (20 mg) was homogenized in 8 volumes of cell lysis buffer (20 mM Tris-HCL pH 7.5, 150 mM NaCl, 1 mM Na_2_EDTA, 1 mM EGTA, 1% Triton, 2.5 mM sodium pyrophosphate, 1 mM Na_3_VO_4_, 1 μg/mL of leupeptin, 1 mM β-glycerophosphate; Cell Signaling Technologies) containing a protease inhibitor cocktail (Roche Applied Sciences). Samples were centrifuged at 13,000 rpm for 45 minutes at 4 °C. The supernatant was collected, and protein concentration was measured using the DC Assay (Bio-Rad laboratories Inc). Equal amounts of protein (20 μg) were diluted in loading buffer (10% glycerol, 0.08 M SDS, 5% β-mercaptoethanol and 0.05 M Tris pH 6.8), boiled at 90 °C for 5 minutes, and loaded onto a 12% polyacrylamide gel. After electrophoresis at 100 V for approximately 2 hours the proteins were transferred onto a methanol-activated polyvinylidene difluoride membrane, at 0.3 A for 1 hour. The membranes were blocked in 5% bovine serum albumin (BSA) in TBS-T at room temperature for 1 hour and incubated with the primary antibody directed against 4HNE (ab46545, Abcam; 1:1000), TOP2β (MA5-24310, ThermoFisher, 1:1000) or against GAPDH (MAB374, Millipore; 1:2500), in 5% BSA in TBS-T overnight at 4 °C. The next day, the membranes were washed and incubated with a species-specific secondary antibody for 1 hour at room temperature. Immunoreactive bands were revealed using enhanced chemiluminescence substrate (PerkinElmer, Inc.) in an Amersham Imager 600. The ImageLab software was used to determine the optical density of each band. For 4HNE immunoreactivity, the pixel intensity of the entire lane was analyzed by densitometry. Immunoblots were stripped and re-probed with GAPDH as a loading control. All values were normalized by GAPDH and expressed as relative values compared to Wt. Each experiment was repeated a minimum of two times.

### Statistics and reproducibility

The software GraphPad Prism version 10 (La Jolla, USA) was utilized for statistical analyses. The D’Agostino and Pearson omnibus normality test was used to assess normal distribution of the data. Outliers were excluded using the ROUT method (Q = 1%). Graphed data is presented as mean values ± SD and two-tailed t-tests were performed for the two-group comparisons that obeyed assumptions of parametric statistics. For data which had unequal variances, Welch’s correction was applied. For data that did not obey the assumptions of parametric statistics, we used the non-parametric Mann-Whitney test. No multiple comparison corrections were performed. Significance was set to p < 0.05. Details of replicates for each experiment can be found in each methods subsection.

### Reporting summary

Further information on research design is available in the Nature Portfolio Reporting Summary linked to this article.

### Supplementary information

Supplemental Information

Description of Additional Supplementary Materials

Supplementary Data 1

Reporting Summary

## Data Availability

Data is available upon reasonable request, numerical source data for graphs in the manuscript can be found in the supplementary data file (Supplementary Source Data 1).
