# Supplementary material for: Early oxidative stress and DNA damage in Aβ-burdened hippocampal neurons in an Alzheimer’s-like transgenic rat model
Source: Commun Biol. 2024 Jul 14;7:861. doi: 10.1038/s42003-024-06552-4 (PMC11247100; doi:10.1038/s42003-024-06552-4)
Supplement: Supplementary file 1 — Supplemental Information [file 42003_2024_6552_MOESM1_ESM.pdf]

Early oxidative stress and DNA damage in Aβ-burdened hippocampal neurons in an Alzheimer's-like transgenic rat model

Morgan K. Foret[1], Chiara Orciani[2], Lindsay Welikovitch[2], Chunwei Huang[1], A. Claudio Cuello[1,2,3,4], Sonia Do Carmo[1*]*

[1]Department of Pharmacology and Therapeutics, McGill University, 3655 Promenade Sir William Osler, Montreal, Quebec H3G 1Y6, Canada.

[2]Department of Neurology and Neurosurgery, McGill University, Montreal, Canada.

[3]Department of Anatomy and Cell Biology, McGill University, Montreal, Canada.

[4]Department of Pharmacology, Oxford University, Oxford OX13QT, UK.

*To whom correspondence should be addressed

Email: sonia.docarmo@mcgill.ca ; claudio.cuello@mcgill.ca

This section includes:

- **Figure S1.** Expression of additional genes related to oxidative stress showing a trend to higher values.
- **Figure S2.** Image analysis methods.
- **Figure S3.** Increased protein levels of 4HNE in the hippocampus of McGill-R-Thy1-APP Tg rats with iAβ-burdened neurons.
- **Figure S4.** RNase pre-treatment for 8-oxo-dG assessment.
- **Figure S5.** DCF Assay.
- **Figure S6.** Original Western blots showing increased protein levels of TOP2β in hippocampal homogenates from Tg rats.
- **Table S1.** Housekeeping genes for RT^2 Rat Oxidative Stress Profiler PCR Array.
- **Table S2.** Expression of oxidative stress-related genes in hippocampal neurons (RT^2 Rat Oxidative Stress Profiler PCR Array).
- **Table S3.** List of primary antibodies and dilutions for IHC and IF experiments.
- **Table S4.** List of secondary antibodies and dilutions for IHC and IF experiments.
- **Table S5.** Primer sequences for quantitative real-time PCR (hippocampal homogenates).

SUPPLEMENTAL FIGURES

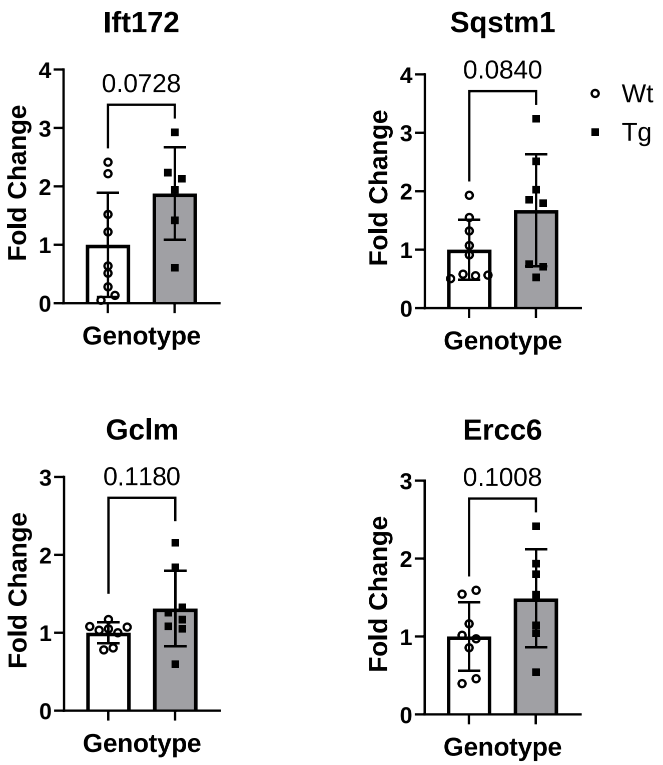

Figure S1. Expression of additional genes related to oxidative stress showing a trend to higher values. Expression of genes in iAβ-burdened Tg hippocampal neurons as compared to Wt neurons including *Ift172*, *Sqstm1*, *Gclm and Ercc6*. Fold changes were normalized to Wt expression. n = 9 (Wt), n = 8 (Tg). Error bars indicate SD.

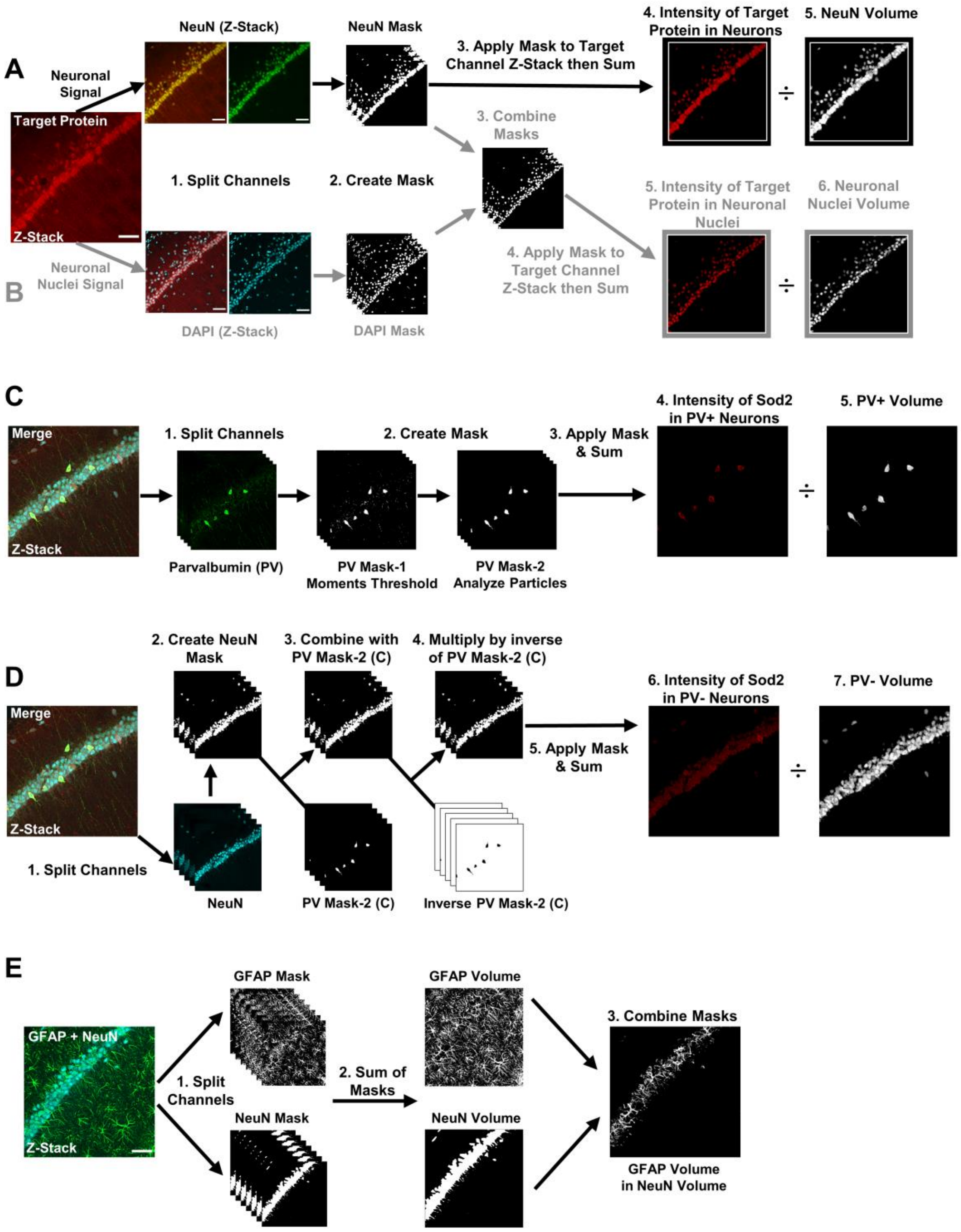
A
Neuronal Signal
NeuN (Z-Stack)
NeuN Mask
3. Apply Mask to Target Channel Z-Stack then Sum
4. Intensity of Target Protein in Neurons
5. NeuN Volume
Target Protein
Z-Stack
1. Split Channels
2. Create Mask
3. Combine Masks
5. Intensity of Target Protein in Neuronal Nuclei
6. Neuronal Nuclei Volume
4. Apply Mask to Target Channel Z-Stack then Sum
B
Neuronal Nuclei Signal
DAPI (Z-Stack)
DAPI Mask
C
Merge
Z-Stack
1. Split Channels
2. Create Mask
3. Apply Mask & Sum
4. Intensity of Sod2 in PV+ Neurons
5. PV+ Volume
Parvalbumin (PV)
PV Mask-1 Moments Threshold
PV Mask-2 Analyze Particles
D
Merge
Z-Stack
2. Create NeuN Mask
3. Combine with PV Mask-2 (C)
4. Multiply by inverse of PV Mask-2 (C)
5. Apply Mask & Sum
6. Intensity of Sod2 in PV- Neurons
7. PV- Volume
1. Split Channels
NeuN
PV Mask-2 (C)
Inverse PV Mask-2 (C)
E
GFAP + NeuN
Z-Stack
GFAP Mask
NeuN Mask
1. Split Channels
2. Sum of Masks
GFAP Volume
NeuN Volume
3. Combine Masks
GFAP Volume in NeuN Volume

Figure S2: Image analysis methods.

A. Quantifying target protein fluorescence intensity in CA1 and subiculum neurons using NeuN. ImageJ macro work-flow using z-stack image files. **(1)** Channels were split and a NeuN mask **(2)** was generated as follows: the NeuN z-stack was processed using despeckle and a z-projection of the average intensity (Fancc, GR) or maximum intensity (SOD2, XPD) was obtained. The threshold method Default (Fancc), Yen (GR), or Moments (XPD) was applied to obtain lower and upper threshold values. The raw NeuN z-stack was then despeckled twice and these threshold values were applied to the entire stack to generate a binary NeuN z-stack which was divided by 255 to obtain pixel values of 0 and 1. **(3)** This NeuN binary z-stack mask was multiplied by the z-stack of the target protein channel and summed to solely obtain **(4)** the fluorescence intensity within the neuronal volume. The NeuN volume **(5)** was obtained by taking the summed projection of the binary NeuN z-stack mask in step 2. Finally, fluorescence intensity was divided by the volume to obtain a mean fluorescence intensity.

B. Quantifying target protein fluorescence intensity in CA1 and subiculum neuronal nuclei using DAPI and NeuN similar to the process in A. The process for generating a NeuN binary mask was as described in A steps (1) and (2). **(1)** Channels were split and a DAPI mask **(2)** was generated as follows: the DAPI z-stack was processed using despeckle and a z-projection of the average intensity was obtained. The threshold method Default was applied to obtain lower and upper threshold values. The raw DAPI z-stack was then despeckled twice and these threshold values were applied to the entire stack to generate a binary DAPI z-stack which was divided by 255 to adjust the pixel values to 0 and 1. **(3)** This DAPI binary z-stack mask was then multiplied by the NeuN binary z-stack to obtain a binary z-stack mask of neuronal nuclei. **(4)** This neuronal nuclei binary z-stack mask was then multiplied by the z-stack of the target protein channel and summed to solely obtain **(5)** the fluorescence intensity within the neuronal nuclei volume. The neuronal nuclei volume **(6)** was obtained by taking the summed projection of the binary z-stack mask in step 3. Finally, fluorescence intensity was divided by the volume to obtain a mean fluorescence intensity.

C. Quantifying target protein fluorescence (specifically SOD2) in PV+ neurons of CA1 and subiculum. **(1)** Channels were split and a PV (parvalbumin) mask **(2)** was generated as follows: the PV z-stack was processed using despeckle and a z-projection of the maximum intensity was obtained. The threshold method Li was applied to obtain lower and upper threshold values. The raw PV z-stack was then despeckled twice and these threshold values were applied to the entire stack to generate a binary PV z-stack. Analyze particles (250 pixels^2) was then applied and the mask was divided by 255 to obtain pixel values of 0 and 1. **(3)** This PV binary z-stack mask was then multiplied by the z-stack of the target protein channel (SOD2) and summed to solely obtain **(4)** the fluorescence intensity within the PV neuronal volume. The PV volume **(5)** was obtained by taking the summed projection of the binary PV z-stack mask in step 2. Finally, fluorescence intensity was divided by the volume to obtain a mean fluorescence intensity.

D. Quantifying target protein fluorescence (specifically Sod2) in PV- neurons of CA1 and subiculum. **(1)** Channels were split and a NeuN mask **(2)** was generated as described in A. **(3)** This NeuN binary z-stack mask was then combined with the PV binary mask generated in C. This combined mask including NeuN and PV was then multiplied by the inverse of the PV mask to generate a mask that excluded PV+ neurons **(4)**. **(5)** This modified NeuN binary z-stack designed to exclude PV+ neurons was then multiplied by the z-stack of the target protein channel (SOD2) and summed to solely obtain **(6)** the fluorescence intensity within the NeuN neuronal volume excluding PV+ neurons. The volume of this modified NeuN mask **(7)** was obtained by taking the summed projection of the binary z-stack mask in step 4. Finally, fluorescence intensity was divided by the volume to obtain a mean fluorescence intensity.

E. Assessing GFAP-positive processes in the vicinity of CA1 and subiculum neurons. **(1)** Channels were split and masks were generated for NeuN (z-projection maximum intensity, despeckle, triangle threshold, binary, fill holes, erode twice, divide by 255 to generate pixel values of 0 and 1 – for the total z-stack area multiply by the number of z-stacks) and for GFAP (gaussian blur with a sigma of 1, threshold applied to each image in stack (obtain threshold value using a mean threshold applied to the average z-stack), binary, divide by 255 to generate pixel values of 0 and 1). **(2)** Take the sum the masks, which represents the total neuronal (NeuN+) and astrocytic (GFAP+) areas. **(3)** To obtain the area of GFAP immunoreactivity in neuronal regions, combine the summed NeuN and GFAP masks by multiplying them (therefore only areas containing both NeuN and GFAP with pixel values of 1 will remain).

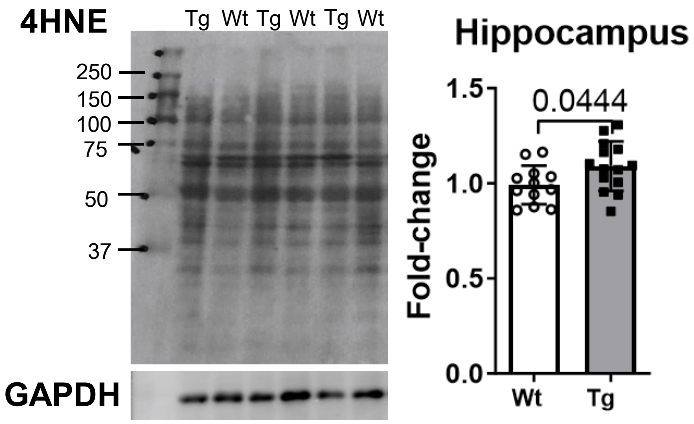

Figure S3: Increased protein levels of 4HNE in the hippocampus of McGill-R-Thy1-APP Tg rats with iAβ-burdened neurons. Representative images (left) and quantification (right) of 4HNE immunoreactivity normalized to GAPDH levels in Wt as compared to Tg hippocampal homogenates as determined by Western blotting. Fold changes were normalized to Wt expression. n = 12 (Wt), n = 15 (Tg). Error bars indicate SD. Two-tailed t-test, $*p < 0.05$.

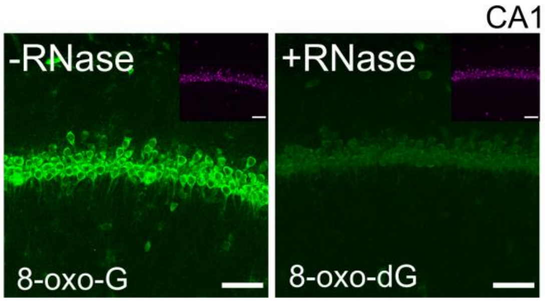

Figure S4. RNase pre-treatment for 8-oxo-dG assessment. RNase pre-treatment diminishes 8-oxo-G immunoreactivity and reveals 8-oxo-dG immunoreactivity. Comparison of pre-treatment without or with RNase, showing 8-oxo-G in green and NeuN as an inset (magenta) using the same imaging and analysis settings. Scale bar represents 50 µm.

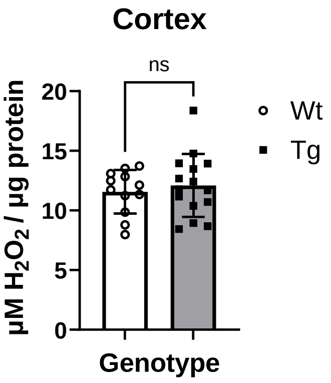

Figure S5. DCF Assay. Quantification of fluorescence signal from cortical homogenates. Signal indicates the general redox status of the samples. n = 12 (Wt), n = 15 (Tg). Error bars represent SD, two-tailed t-test, ns = non-significant.

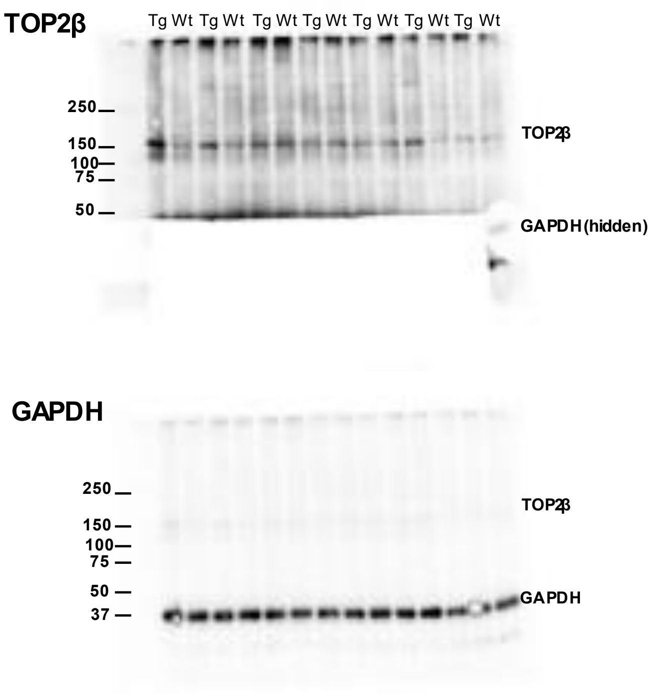

Figure S6. Original Western blots showing increased protein levels of TOP2β in hippocampal homogenates from Tg rats. Representative images of TOP2β immunoreactivity normalized to GAPDH levels in Wt as compared to Tg hippocampal homogenates as determined by Western blotting.

SUPPLEMENTAL TABLES

Table S1. Housekeeping genes for RT^2 Rat Oxidative Stress Profiler PCR Array.

Abbreviation	Full name
Actb	β-Actin
B2m	β2 microglobulin
Hprt1	Hypoxanthine phosphoribosyltransferase 1
Ldha	Lactate dehydrogenase A
Rplp1	Ribosomal protein, large, P1

Table S2. Expression of oxidative stress-related genes in hippocampal neurons (RT^2 Rat Oxidative Stress Profiler PCR Array). n/a indicates genes where qRT-PCR amplification was not efficient in enough samples from both groups (19 of the 84 genes were not assessed). $^+$Mann Whitney test, otherwise t-tailed t-test. $^{\%}$Welch's correction applied for unequal variances. Values in **bold** were found to be significantly different between Wt and Tg.

Gene	Fold-change	P value	Gene	Fold-change	P value
Alb	0.80	0.5892	*Mb*	n/a	n/a
Als2	1.11	0.4903	*Mpo*	n/a	n/a
Aox1	n/a	n/a	*Ncf1*	n/a	n/a
Apc	0.98	0.8866	*Ncf2*	0.86	0.6859
Apoe	1.60	0.3213$^+$	*Ngb*	1.07	0.8676
Cat	1.10	0.7371	*Nos2*	1.63	06623$^+$
Ccl5	n/a	n/a	*Nox4*	n/a	n/a
Ccs	1.24	0.6034	*Noxa1*	n/a	n/a
Ctsb	1.38	0.4807	*Noxo1*	n/a	n/a
Cyba	n/a	n/a	*Nqo1*	0.98	0.9557
Cygb	1.03	0.9061	*Nudt1*	1.04	0.8607
Dhcr24	1.72	0.2325$^{\%}$	*Park7*	1.33	0.3312
Dnm2	1.65	0.2114	*Prdx1*	0.99	0.8902
Duox1	2.02	0.3205$^{\%}$	*Prdx2*	1.02	0.9626$^+$
Duox2	1.14	0.6058$^+$	*Prdx3*	1.36	0.1910
Ehd2	0.82	0.6095$^+$	*Prdx4*	1.07	0.7930
Epx	0.51	0.4634$^+$	*Prdx5*	1.43	0.2359$^+$
Ercc2	**1.52**	**0.0365**	*Prdx6*	1.57	0.1529
Ercc6	1.49	0.1008	*Prnp*	1.74	0.1996$^+$
Fancc	**2.47**	**0.0392**	*Psmb5*	1.19	0.3275
Fmo2	1.26	0.6620$^+$	*Ptgs1*	3.03	0.0976
Fth1	1.11	0.3367	*Ptgs2*	1.07	0.9626$^+$
Gclc	0.94	0.7249	*Rag2*	n/a	n/a
Gclm	1.31	0.1180$^{\%}$	*Scd1*	1.90	0.1404
Gpx1	1.20	0.6994	*Sels*	1.12	0.3704$^+$
Gpx2	n/a	n/a	*Sepp1*	0.74	0.5414$^+$
Gpx3	1.36	0.3864	*Serpinb1b*	0.83	0.5602
Gpx4	1.62	0.3008	*Slc38a1*	1.13	0.6425
Gpx5	n/a	n/a	*Slc38a5*	n/a	n/a
Gpx6	2.72	0.1388$^+$	*Sod1*	0.98	0.9213
Gpx7	1.56	0.3704	***Sod2***	**1.44**	**0.0351**
Gsr (GR)	**1.77**	**0.0200**	*Sod3*	0.97	0.9324
Gstk1	1.09	0.7669	*Sqstm1*	1.68	0.0840
Gstp1	1.28	0.1552	*Srxn1*	1.24	0.3084$^{\%}$
Hba-a2	1.59	0.5494	*Tpo*	n/a	n/a
Hmox1	0.93	0.3823$^+$	*Txn1*	0.89	0.7486
Hspala	n/a	n/a	*Txnip*	2.04	0.5218
Idh1	**1.74**	**0.0011**$^+$	*Txnrd1*	1.49	0.1793
Ift172	1.88	0.0728	*Txnrd2*	1.39	0.1299
Krt1	n/a	n/a	*Ucp2*	1.51	0.4439
LOC367198	n/a	n/a	*Ucp3*	n/a	n/a
Lpo	0.50	0.4668	*Vim*	n/a	n/a

Table S3: List of primary antibodies and dilutions for IHC and IF experiments.

Target	Source	Category Number	Species and Clonality	Dilution
4HNE	Abcam	Ab46545	Rabbit polyclonal	1:250
8-oxo-dG	Trevigen/R&D	4354-MC-050	Mouse monoclonal	1:500
Fancc	LS-Bio	LS-C331704	Rabbit polyclonal	1:100
γH2AX	Abcam	Ab26350	Mouse monoclonal	1:1000
GFAP	Novus	SPM507	Mouse monoclonal	1:2000
GR	ThermoFisher	PA5-29945	Rabbit polyclonal	1:250
Idh1	Abcam	ab172964	Rabbit monoclonal	1:100
NeuN	EMD Merck Millipore	MAB377X	Mouse monoclonal (AF 488)	1:500
NeuN	EMD Merck Millipore	ABN90P	Guinea pig polyclonal	1:1000
NeuN (with γH2AX)	Abcam	ab177487	Rabbit monoclonal	1:2500
Aβ	Medimabs	McSA1	Mouse monoclonal	1:1000
Parvalbumin	EMD Merck Millipore	MAB1572	Mouse monoclonal	1:5000
SOD2	Abcam	ab68155	Rabbit monoclonal	1:100
XPD	Abcam	ab111596	Rabbit polyclonal	1:500

Table S4: List of secondary antibodies and dilutions for IHC and IF experiments.

Fluorophore Conjugate	Source	Category Number	Species and Clonality	Dilution
Not Applicable	In-house	Not Applicable	Rabbit-anti-Mouse	1:25
Alexa Fluor 488	ThermoFisher	A-11029	Goat-anti-Mouse Polyclonal Highly Cross-Adsorbed	1:800
Alexa Fluor 568	ThermoFisher	A-11036	Goat-anti-Rabbit Polyclonal Highly Cross-Adsorbed	1:800
Alexa Fluor 647	ThermoFisher	A-21450	Goat-anti-Guinea pig Polyclonal Highly Cross-Adsorbed	1:800

Table S5. Primer sequences for quantitative real-time PCR (hippocampal homogenates).

Gene	Forward Primer 5' – 3'	Reverse Primer 5' – 3'	Amplicon Length (bp)
DNA repair-related genes			
Ape1	CGTTGGGAGGCAGCGTAGTA	CTTCTTGGTCTCTGGCTCGG	138
Brca1	TTAACAGGGCGGTCTTGCTT	GGCTGGCACTTTAGGGGATT	117
Cdk5	GTATCCCAGTCCGCTGCTAC	CTGTTCCTCAGTCGGTGTCC	224
Ercc3	CTGCCAGAAGCAAATGTCCTC	CTGCGACCATCCCTTTCTTG	109
Ercc6	TTGCAAACAGAATAACCTGCG	GCAGCGAAAGGCTCATCTTG	242
Fen1	CGCTGGTAGGAAGAAGCCATT	ACCCTGACGAACAGCAATCA	182
Ogg1	CTGGGCCTCTATTCCGTGTC	TTTGCTCCCTCCACCGGAAG	86
Parp1	ACCACGCACAATGCCTATGA	AGTCTCCGGTTGTGAAGCTG	107
Pcna	TGCAGATGTACCCCTTGTTGT	CATCTTCGATCTTGGGAGCCA	83
Pnkp	CCAAAGTCGGGCTAGGTACA	CATGTCTGATACCGGCGCA	148
Polβ	AATGAGTACACCATCCGCCC	GCGTCATTCACTCCTGTCCT	132
Prkdc	TCACAAGAGGAGAAAGTGGCT	CTGTACGGTTAGCTCTGCTGT	136
Sirt1	TGTGCAGTGGAAGGAAAGCAA	GTTGCAAAGGAACCATGACAC	187
Sirt3	GGGCTTGAGAGAGCATCTGG	ACAACGCCAGTACAGACAGG	167
Sirt6	TTATGCAGCAGGGTTGTCGC	CTGGTGGGTCGAATATCTCGG	71
Rad51	CGAAGTGTGTTTGAGCCGTG	CTGCATTTGCTTCAAGCTGC	127
Rpa	CCATAATGCAGCAGGGAGATAC	AGGAAAGCGTGTTCAACCCAT	129
Tdp1	CTGTGGAAAATGTGCGGACC	AGCCACCTCTGTTTCTCAGC	91
Xrcc1	GGAACAGTCAGAAGGACGGG	ATCTGTGGAGCCTGCATACG	145
Xrcc4	AGTGGAGTCACTGAGAGGTCA	TATCGTCCCCTCCCAAGACA	111
Xrcc5	TTTGAGGAAGCGAGTCTCCAG	AAGGCCTTCCAGGAAACTGT	162
Xrcc6	CAGCCCGAAAGAATGTCTCC	TCATCGGCGTAAGGGAGGAA	124
Early response genes (ERGs)			
Grin2b	AACCCTCGTGGCCAGCA	CAGCTAGTCGGCTCTCTTGGTT	65
CamkIIα	GCCTACATCCGCATCACTCA	CTGGCCTGGTCCTTCAATGG	163
CamkIIβ	TTCCGACAGCACCAACACAA	CACTGATAGGGGTCCCTCGG	121
BdnfIV	CGCCATGCAATTTCCACTATCAATAATTTAAC	CTTTTTCAGTCACTACTTGTCAAAGTAAC	200
c-fos	CTCAGTTGCTAGCTGCAATCG	CCCCCTCCAGTTTCTCTGTT	113
Homer1a	CGCAGGAGAAGATGGAACTGA	TTTCTGGTGTTAAAGGAGACTGAAGA	81
Zif268(Egr1)	CTTGGATGGGAGGTCTTCAC	CGAATCGGCCTCTATTTCAA	145
Housekeeping (control) genes			
Hprt	CAGGCCAGACTTTGTTGGAT	TCCACTTCCGCTGATGACAC	114
Gapdh	TGATGGGTGTGAACCACGAG	TCATGAGCCCTTCCACGATG	132
